# An Evaluation of the Complement-Regulating Activities of Human Complement Factor H (FH) Variants Associated With Age-Related Macular Degeneration

**DOI:** 10.1167/iovs.63.12.30

**Published:** 2022-11-29

**Authors:** Robyn M. Biggs, Elisavet Makou, Scott Lauder, Andrew P. Herbert, Paul N. Barlow, Suresh K. Katti

**Affiliations:** 1 Gemini Therapeutics, Inc., Cambridge, Massachusetts, United States; 2 School of Chemistry, University of Edinburgh, Edinburgh, United Kingdom; 3 School of Biological Sciences, University of Edinburgh, Edinburgh, United Kingdom

**Keywords:** age-related macular degeneration, complement, factor H, alternative pathway, factor H missense variants, therapy

## Abstract

**Purpose:**

Factor H (FH, encoded by *CFH*) prevents activation of the complement system's alternative pathway (AP) on host tissues. FH impedes C3 convertase (C3bBb) formation, accelerates C3bBb decay, and is a cofactor for factor I (FI)–catalyzed C3b cleavage. Numerous *CFH* variants are associated with age-related macular degeneration (AMD), but their functional consequences frequently remain undetermined. Here, we conduct functional comparisons between a control version of FH (not AMD linked) and 21 AMD-linked FH variants.

**Methods:**

Recombinantly produced, untagged, full-length FH versions were assayed for binding to C3b and decay acceleration of C3bBb using surface-plasmon resonance, FI-cofactor activity using a fluorescent probe of C3b integrity, suppression of C5b-9 assembly on an AP-activating surface, and inhibition of human AP-mediated lysis of sheep erythrocytes.

**Results:**

All versions were successfully purified despite below-average yields for Arg2Thr, Arg53Cys, Arg175Pro, Arg175Gln, Ile221Val, Tyr402His, Pro503Ala, Arg567Gly, Gly1194Asp, and Arg1210Cys. Compared to control FH, Arg2Thr, Leu3Val, Ser58Ala, Asp90Gly, Asp130Asn, Gln400Lys, Tyr402His, Gly650Val, Ser890Ile, and Thr965Met showed minimal functional differences. Arg1210C, Arg53His, Arg175Gln, Gly1194Asp, Pro503Ala, Arg53Cys, Arg576Gly, and Arg175Pro (in order of decreasing efficacy) underperformed, while Ile221Val, Arg303Gln, and Arg303Trp were “marginal.” We newly identified variants toward the center of the molecule, Pro503Ala and Arg567Gly, as potentially pathogenic.

**Conclusions:**

Our approach could be extended to other variants of uncertain significance and to assays for noncanonical FH activities, aiming to facilitate selection of cohorts most likely to benefit from therapeutic FH. This is timely as recombinant therapeutic FH is in development for intravitreal treatment of AMD in patients with reduced FH functionality.

Age-related macular degeneration (AMD) is the leading cause of irreversible blindness in high-income countries.^1^ It is estimated to affect over 200 million people globally in 2022 and predicted to afflict as many as 290 million by 2040.^1^ Dysregulation of the complement system has been widely linked to the development of AMD. For example, macular drusen—the accumulation of which is key to vision loss in AMD^2^—are enriched with complement proteins.^3^ A further analysis of tissue from 45 AMD donors provided convincing evidence for complement deposition in high-risk macular drusen (i.e., in central macula, under the fovea).^4^

Multiple genome-wide association studies implicate the *CFH* gene that encodes the complement regulator factor H (FH), a soluble plasma glycoprotein, in the development of AMD,^5^^–^^7^ and some AMD-linked *CFH* variants are associated with early onset or a severe form of the disease.^8^^–^^11^ In addition, numerous retrospective studies have identified various single-nucleotide polymorphisms located throughout *CFH* that potentially contribute to AMD.^8^^,^^12^^–^^16^ These data suggest that *CFH* gene therapy, or administration of the glycoprotein itself,^17^ could yield therapeutic benefit. However, predictions of phenotypic consequences of genetic variation in *CFH* have proved unreliable (e.g., in the case of atypical hemolytic uremic syndrome [aHUS]–linked FH variants),^18^ supporting the need for experimental verification of AMD-linked variants.

Within the complement system, FH regulates cleavage of complement component 3 (C3) to its activated form (C3b), which in turn triggers the alternative pathway (AP) of the complement system.^19^^,^^20^ Crucially, FH inhibits the amplification of C3b production on self-surfaces and in fluid phase but avoids doing so on hazardous cells, cellular debris, and foreign particles that need to be cleared. FH derives its selectivity by recognizing markers, such as glycosaminoglycans and sialic acids, which are typically exclusive to healthy self-surfaces.^21^^,^^22^ FH competes with complement factor B (FB) for binding to C3, thereby inhibiting formation of C3 convertase (C3bBb) that otherwise, in a positive-feedback loop, amplifies cleavage of C3 to C3a and additional C3b. FH also accelerates irreversible decay of C3bBb and is a cofactor for complement factor I (FI) that cleaves C3b to interrupt its amplification loop and to generate iC3b; iC3b is an opsonin that contributes to complement-mediated clearance of cellular debris.^23^

The canonical activity of FH can, therefore, be measured by evaluating its C3b binding, C3bBb decay accelerating activity (DAA), and FI-cofactor activity (CA) as well as assaying its abilities to prevent deposition of components of the terminal pathway of complement on activating surfaces and to protect red blood cells from hemolysis. These functions map to the four N-terminal and two C-terminal modules of the 20 complement control protein modules (CCPs) that comprise the FH molecule.^24^ A crystal structure of mini-FH (an engineered version of FH in which CCPs 1–4 are flexibly linked directly to CCPs 19–20) and catalytically inactive FI, in ternary complex with C3b, indicated that CCPs 2–4 and CCPs 19–20 interact with separate sites on C3b, while CCPs 2 and 3 also bind FI (Fig. 1).^25^ Moreover, CCPs 19–20 additionally recognize self-surface specific markers as well as iC3b and C3d already deposited on the cell surface.^26^ Binding sites on other CCPs, notably for C-reactive protein, complement receptor 3, and apoE, mediate other FH activities, including some unrelated to its canonical function of AP suppression.^27^

**Figure 1.**
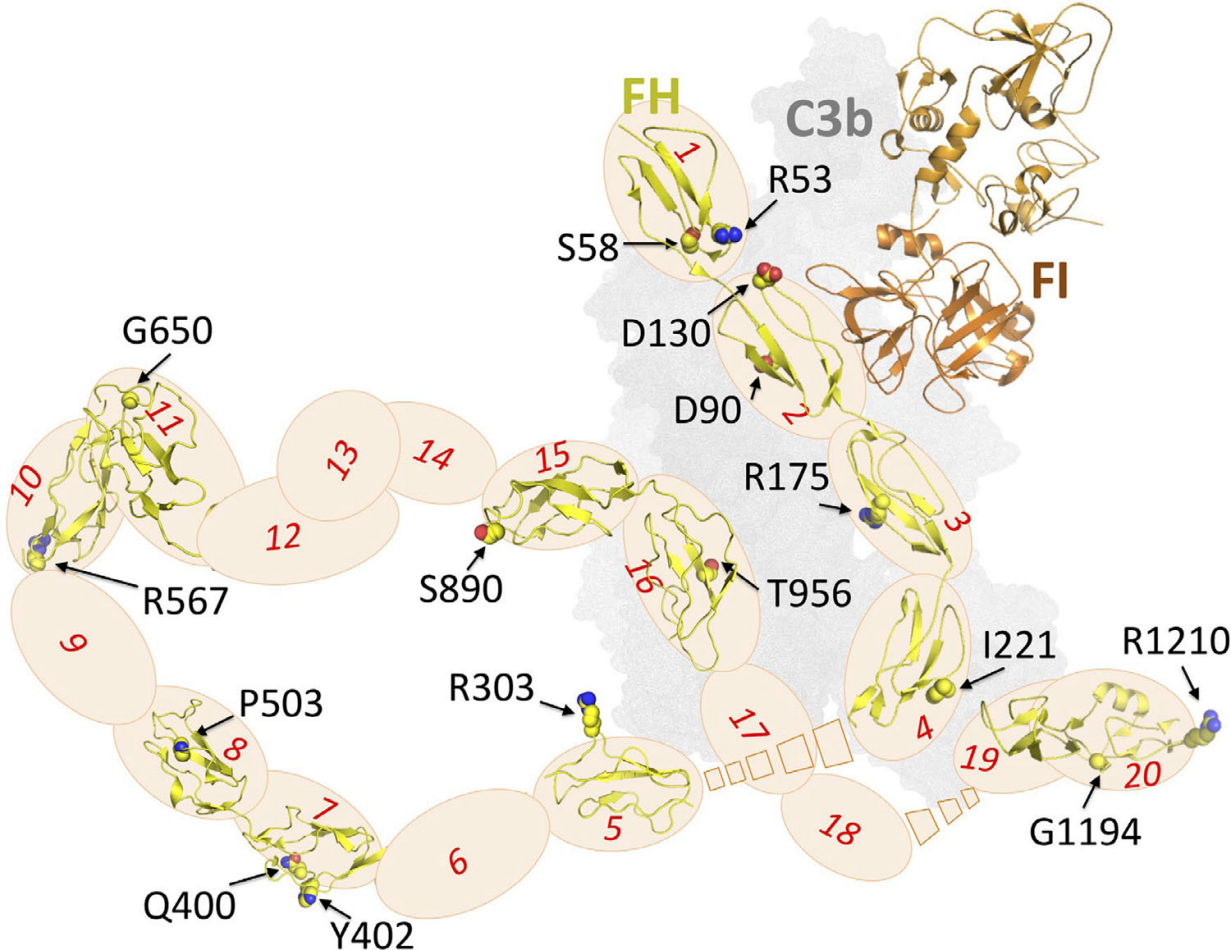
Schematic diagram of a FH:FI:C3b complex, indicating the amino acid residues substituted in the FH variants produced for this study. The C3b surface is represented by a *gray* *shadow*, FI is shown as a *cartoon*, and FH as a series of numbered *ovals*, signifying its 20 CCP modules and cartoon-style representations of those CCPs harboring substituted residues (labeled). The schematic is based on manually concatenating (with no attempt at computational model building) the following Protein Data Bank (PDB) entries: 5O32 (C3b:mini-FH [i.e., CCPs 1–4 linked to CCPs 19–20]:FI),^25^ 2UWN (FH CCPs 6–8),^33^ 4B2R (FH CCPs 10–11), 4B2S (FH CCPs 11–12),^34^ 2KMS (FH CCPs 12–13),^35^ and 1HFI (FH CCPs 15–16).^36^ Note that the positions of CCPs 5–18, relative to CCPs 1–4 and CCPs 19–20 that are bound to C3b in PDB 5O32, are unknown (indicated by *broken boxes*)—some of these CCPs may or may not contact C3b. See also Figure 7B and Supplementary Figure S8. D, aspartic acid; G, glycine; I, isoleucine; P, proline; Q, glutamine; R, arginine; S, serine; T, threonine; Y, tyrosine.

In the current study, a set of *CFH* variants that are among the most prevalent and consequential in the AMD population was selected, and a panel of biochemical and cell-based assays was used to compare the canonical, complement-regulating properties of the encoded proteins produced in human cell culture. We further attempted to rank the variants from most to least functionally impacted by combining all of the activity data with protein yields to provide a composite measure of deleteriousness that we termed a *combined efficacy score* (CES). This aims to provide experimental context to the potential application of FH-based therapeutics in selected individuals possessing specific AMD-linked variants.

## Materials and Methods

### Production, Purification, and Strategy for Characterization of FH Variants

The 21 FH variants of interest and a control version of FH were produced using standard methods from human cell culture, as previously described^28^—all variants were produced in an identical manner from recombinant DNA constructs that were verified by Sanger sequencing. For a control, we chose the *CFH*-encoded glycoprotein with primary-sequence ID P08603 in UniProtKB, which has no specific disease relevance. Following purification by affinity chromatography (using a resin proprietary to Gemini Therapeutics—recombinant FH [GEM103] (Cambridge, Massachusetts, United States) purified with this resin was previously shown to be equivalent in activity to serum-derived FH),^28^ the homogeneity and approximate molecular weights of the protein products were verified by sodium dodecyl sulfate polyacrylamide gel electrophoresis (see Supplementary Methods I for details). We then performed five functional assays (described below) to assess the ability of each FH variant to suppress C3b production and inhibit the AP of the complement system.

#### Measurement of C3b Binding

To compare how tightly each FH variant binds to C3b, we performed surface plasmon resonance (SPR) at 25°C on a Biacore T200 instrument (GE Healthcare, Chicago, IL, USA). In this technique, a solution of each variant is flowed over C3b molecules immobilized on a surface, and the amount of FH variant that binds to the C3b is measured. Thus, 300 response units (RUs) of C3b (Complement Technology, Tyler, TX, USA) were attached, using standard amine coupling, to a Biacore C1 chip (GE Healthcare). A solution containing 250 nM variant FH, or control FH, was injected over the immobilized C3b followed by a dissociation period, after which the sensor chip was regenerated. Each variant was injected in duplicate alongside injections of control FH. Data were analyzed using the Biacore Evaluation software. Confirmatory measurements of equilibrium dissociation constants (*K*_D_) values were performed for selected variants. See Supplementary Methods II for details.

#### Assay of Decay Accelerating Activity

To estimate the ability of each FH variant to accelerate the rate at which the C3 convertase (C3bBb, i.e., a binary complex of C3b and Bb) decays, we used a well-established SPR-based assay^29^ performed at 25°C on a Biacore T200 instrument. In this technique, the dissociation of Bb from surface-immobilized C3bBb is monitored as a function of time. Thus, 2845 RUs of C3b (Complement Technology) were attached, using standard amine coupling, to a Biacore CM5 sensor chip (GE Healthcare), after which C3bBb was assembled on the chip surface as previously described.^28^ Its decay was subsequently monitored in the presence or absence of injected FH. Data were analyzed using the Biacore Evaluation software. See Supplementary Methods III for details.

#### Cofactor Assay

The ability of FH to function as a cofactor for FI in the proteolytic cleavage of C3b was measured using fluorescence spectroscopy.^30^ This assay takes advantage of the fact that the fluorescent molecule 8-anilinonaphthalene-1-sulfonic acid (ANS) binds well to C3b but does not bind tightly to its cleaved products. Thus, FI (purified, from human plasma; Complement Technology) was added to a mixture of FH and C3b (Complement Technology) and then the loss of intact C3b was assessed over time by ANS fluorescence using a SpectraMax M5e (Molecular Devices, San Jose, CA, USA) plate reader. The half-maximal effective concentration (EC_50_) values for FH variant versus control FH were calculated in GraphPad Prism (GraphPad Software, La Jolla, CA, USA) based on analyses of the nonlinear regression of the dose–response curves. See Supplementary Methods IV for details.

#### Inhibition of Alternative Pathway Measured by Terminal Complement Complex (sC5b-9) Immunoassay

A consequence of the actions of FH on C3b and C3 convertase is inhibition of the terminal pathway of complement that is responsible for assembly of the C5b–C9 complex. We used a commercially available assay kit to monitor the extent to which each FH variant suppresses C5b–C9 formation. The AP-specific immunoassay (the Wieslab assay; Svar Life Science, Malmo, Sweden; product code: COMPL AP330) was used to test the ability of FH variants to inhibit soluble (s) C5b-9 formation in the presence of an AP-activating lipopolysaccharide-coated surface. The assay was performed according to the manufacturer's instructions and as described by Seelen et al.,^31^ except human serum was replaced by FH-depleted human serum (Complement Technology) supplemented with FH of interest. Thus, a concentration series of FH variant or control was added to FH-depleted human serum. The EC_50_ values were calculated using GraphPad Prism by interpolation of the curve fits at 50% inhibition.

#### Hemolysis Prevention Assay

A sheep erythrocyte (SE) hemolysis assay allowed assessment of the ability to regulate complement activation at the cell surface. Erythrocytes lyse if complement is dysregulated such that cells are not sufficiently protected by FH. The assay measures lysis prevention by each variant, in comparison to control FH, using fresh human serum (FH depleted and supplemented with the FH of interest) as a source of complement components,^32^ using a SpectraMax M5e (Molecular Devices) plate reader. Protection-against-hemolysis curve fits were generated for each variant and control FH using GraphPad Prism. See Supplementary Methods V for details.

### Combined Efficacy Score

For each variant protein, the results of the above five assays were combined along with an estimate of the relative levels of its secretion, in order to generate a CES. While the CES is by definition a coarse-grained measure of functional activity, it provides a convenient means of ranking FH variants according to their likely impact on the regulation of complement activation in vivo. CES was calculated using the following equation in which control FH has a CES = 100.

$$\begin{array}{c} \begin{array}{c} \mathrm{CES}\\ =100/6\times \sum \;\;\{\;\begin{array}{c} \left(\mathrm{ratio}\;\frac{v}{c}\;C3b\;\mathrm{binding}\right),\left(\mathrm{ratio}\;\frac{c}{v}\;\mathrm{CA}\;EC_{50}\right),\left(\mathrm{ratio}\;\frac{v}{c}\;\mathrm{DAA}\right)\;,\\ \left(\mathrm{ratio}\;\frac{c}{v}\;\mathrm{AP}\;\mathrm{immunoassay}\;EC_{50}\right),\left(\mathrm{ratio}\;\frac{c}{v}\;\mathrm{protein}\;\mathrm{yield}\right)\;,\\ \left(1=\mathrm{no}\;\mathrm{hemolysis}\;\mathrm{or}\;0.5=\mathrm{partial}\;\mathrm{protection}\;\mathrm{or}\;0=\mathrm{hemolysis}\right)\; \end{array} \end{array}\;\;\;\; \end{array}$$

where v = variant, c = control, CA = FI-cofactor assay result, and DAA = decay accelerating activity result; AP immunoassay refers to the result of the AP immunoassay and “no hemolysis” and so on to the results of the assay to measure protection of SE from AP-mediated hemolysis.

## Results

### Choice of Variants

The proteins encoded by 21 *CFH* variants that are among the most prevalent in the AMD population,^8^^,^^12^^–^^16^ along with control FH, were selected for this study. Locations of the substitutions in the mature protein are highlighted in a schematic representation of a ternary complex containing FH, C3b, and FI (Fig. 1).^25^^,^^33^^–^^36^ Table 1 lists the variants and the function (if known) of each affected FH CCP module,^33^^,^^37^ the odds ratio for AMD risk^14^ where known, and the allele frequency in AMD cohorts versus the general population (from the Genome Aggregation Database).

**Table 1.** Allele Frequency of the 21 Most Prevalent AMD-Related FH Variants

FH Variant	CCP Module Affected (Role in C3b, C3d, or Surface Binding)	Odds Ratio for AMD Risk	AMD Allele Frequency (%)	gnomAD Allele Frequency (%)	dbSNP Designation
Control CFH	NA	NA	—	0.67	rs1061170(T)
Arg2Thr	Signal peptide	14.08^*^	0.021	0.002	rs142266551
Leu3Val	Signal peptide	1.06	0.333	0.024	rs139254423
Arg53Cys	1 (C3b binding)	22.54^*^	0.078	0.002	rs757785149
Arg53His	1 (C3b binding)	13.39^*^	0.036	ND	
Ser58Ala	1 (C3b binding)	2.82^*^	0.072	0.025	rs141336681
Asp90Gly	2 (C3b and factor I^†^ binding)	Risk (LOD = 1.22)^*^	0	0.0004	rs1239695899
Asp130Asn	2 (C3b and factor I^†^ binding)	10.0	0.09	0.014	rs147002633
Arg175Gln	3 (C3b and factor I^†^ binding)	1.50^*^	0.34	ND	
Arg175Pro	3 (C3b and factor I^†^ binding)	Not known	0.026	0.0004	rs139360826
Ile221Val	4 (C3b binding)	11.80^*^	0.024	0.002	rs774239374
Arg303Trp	5	12.25^*^	0.016	0.006	rs142937931
Arg303Gln	5	9.47	0.01	0.002	rs766408580
Gln400Lys	7 (GAG binding^‡^)	Not known	0.089	0.012	rs201671665
Tyr402His	7 (GAG binding^‡^)	4.6	0.7	0.33	rs1061170(C)
Pro503Ala	8	Risk (NA)^*^	0.103	0.002	rs570523689
Arg567Gly	10	5.11	0.863	0.0004	rs757756991
Gly650Val	11	1.52	0.073	0.023	rs143237092
Ser890Ile	15	1.03	0.386	2.13	rs515299
Thr956Met	16	1.04	0.275	0.129	rs145975787
Gly1194Asp	20 (C3b or C3d^†^ and cell surface/GAG binding^‡^)	7.41	0.083	0.001	rs761877050
Arg1210Cys	20 (C3b or C3d^†^ and cell surface/GAG binding^‡^)	31.8^*^	0.53	0.015	rs121913059

C3b/d, complement component 3b/d; dbSNP, The Single Nucleotide Polymorphism Database; gnomAD, Genome Aggregation Database; GAG, glycosaminoglycan; LOD, logarithm of the odds; NA, not applicable; ND, not determined.

* Reviewed in Geerlings et al.^14^

† Reviewed in Dunne et al.^37^

‡ Reviewed in Prosser et al.^33^

### A Fivefold Range in Yields of Secreted Proteins

The yield of control FH after purification from 1 L of cell culture was 83 mg. The deployment of equivalent culture conditions and identical purification protocols (producing homogeneous protein samples; see Supplementary Fig. S1A) for all proteins permitted meaningful comparisons of yields that might have a bearing on relative protein production levels in vivo, albeit this represents just a single experiment and was not repeated. The yield for each variant divided by that of control FH ranged from 0.26 for Arg175Pro to 1.3 for Arg53His (see Supplementary Fig. S1B). Despite below-average yields of Arg2Thr, Arg53Cys, Arg175Gln, Arg175Pro, Ile221Val, Tyr402His, Pro503Ala, Arg567Gly, Gly1194Asp, and Arg1210Cys, sufficient quantities of all variants were obtained for a panel of biochemical and cell-biological assays, described below, to be applied.

### Choice of Functional Assays

The intermolecular interactions probed in the five functional assays are summarized in Table 2. Each of the chosen assays assesses all or a subset of the canonical complement pathway regulatory functions of FH (reviewed above). In each assay, in general, the activity of each variant was compared to control FH in a side-by-side pairwise manner in duplicate replicates.

**Table 2.** Summary of Functional Assays Conducted During This Study and the Molecular Interactions of FH Assessed in Each Assay

Assays	Binding to C3b	Competition With FB Binding and/or Displacement of Bb	Recruitment of FI to C3bBb	Recognition of Host Surface
C3b binding	+	−	−	−
DAA	+	+	−	−
Cofactor	+	−	+	−
AP immunoassay	+	+	+	−
Hemolysis	+	+	+	+

#### Impact of AMD-Associated Genetic Variations on C3b Binding

**Figure 2.**
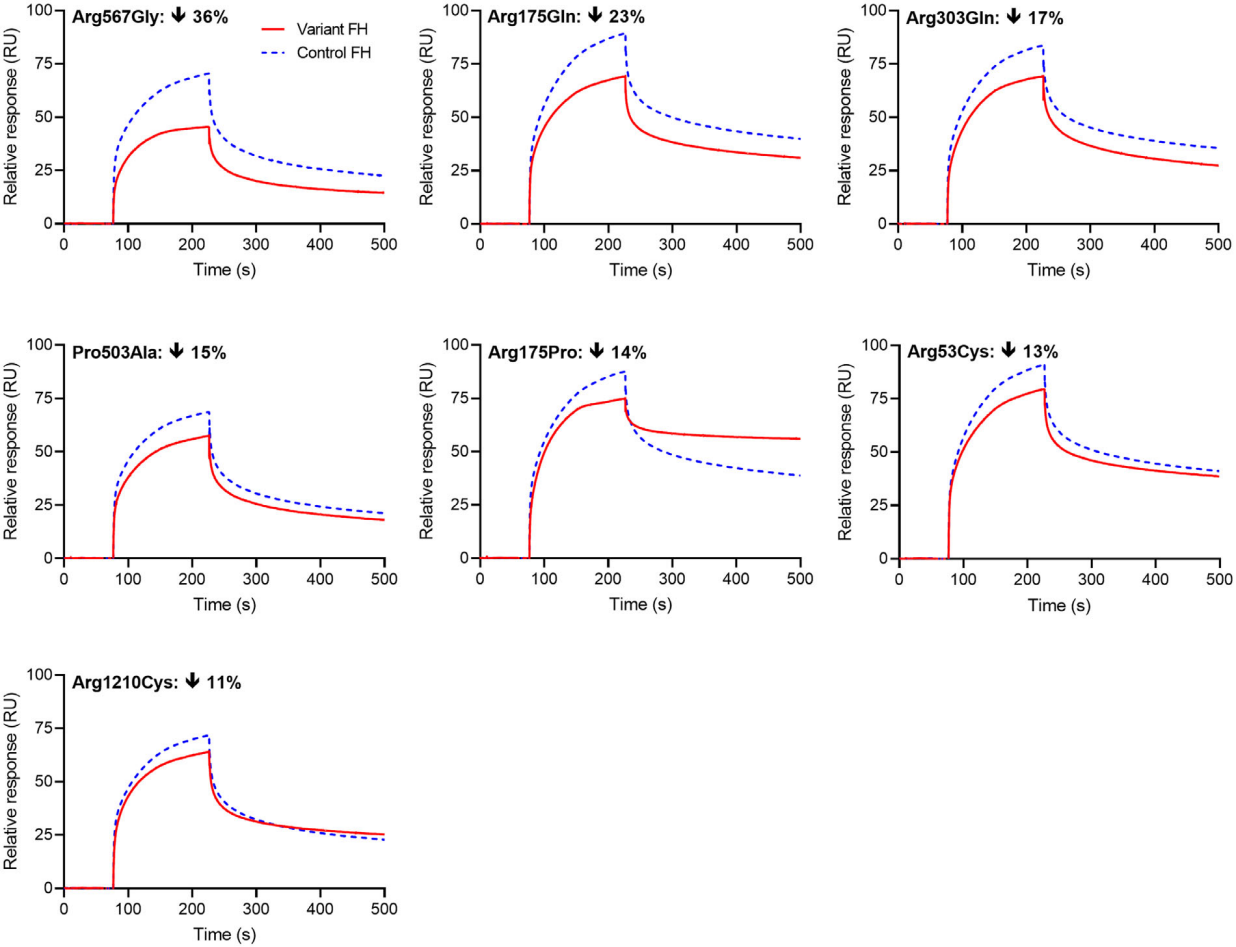
C3b-binding traces for the FH variants that are most impacted by their respective substitutions. Binding of FH to immobilized C3b and then dissociation of FH (upon ceasing injection of FH over the chip) was observed. Each panel presents an overlay of representative traces, obtained in a sequential pairwise fashion, for a variant FH (*red*, *solid*) and control FH (*blue*, *dashed*) (each performed in duplicate). The percentage difference in RUs at binding equilibrium (at 220 seconds) following the injection of variant FH versus control FH is indicated within each panel. See also Supplementary Figure S2. s, seconds.

Binding to C3b is involved in all AP-related functions of FH. All 21 single-substitution variants bound C3b coupled to a SPR chip (Fig. 2 and Supplementary Figs. S2A, S2B). This is unsurprising as FH has at least two binding sites for C3b.^24^ Binding data for single-concentration (250 nM FH) measurements are displayed in Figure 2 for the seven most impacted variants: Arg567Gly, Arg175Gln, Arg303Gln, Pro503Ala, Arg175Pro, Arg53Cys, and Arg1210Cys. Variant Gly1194Asp (in CCP 20) is an outlier that bound more quickly to C3b, and in greater quantities, than control FH—this may be due to dimerization on the chip, a trace of which was observed on the electrophoresis gel (Supplementary Figs. S1A, S2A). Equilibrium *K*_D_s for selected variants were also measured by SPR (Supplementary Table S2). The *K*_D_s of Arg567Gly, Arg175Pro, and Arg53His (the eighth most impacted variant in single-concentration experiments) are two or three times higher than that of control FH. Arg1210Cys has a similar *K*_D_ to control FH but has the lowest maximum binding response of the variants in the table, implying fewer molecules in the sample bind C3b at saturation. In single-concentration experiments, the pairwise comparison of RUs^variant FH^/RUs^control FH^ at binding equilibrium (i.e., at 220 seconds; Supplementary Table S1; plotted in Supplementary Fig. S2C) ranged from 0.65 to 1.27 for the full set. Variants with ratios in the range of 0.9 to 1.2 were regarded to have “normal” function (see Supplementary Table S1 for explanation). Arg2Thr and Leu3Val, with identical post-secretion sequences as control FH, were observed to fall within this range. Binding data for additional variants that were also close to or within the range of values observed for control FH are shown in Supplementary Figure S2B.

#### Impact of AMD-Associated Genetic Variations on DAA

**Figure 3.**
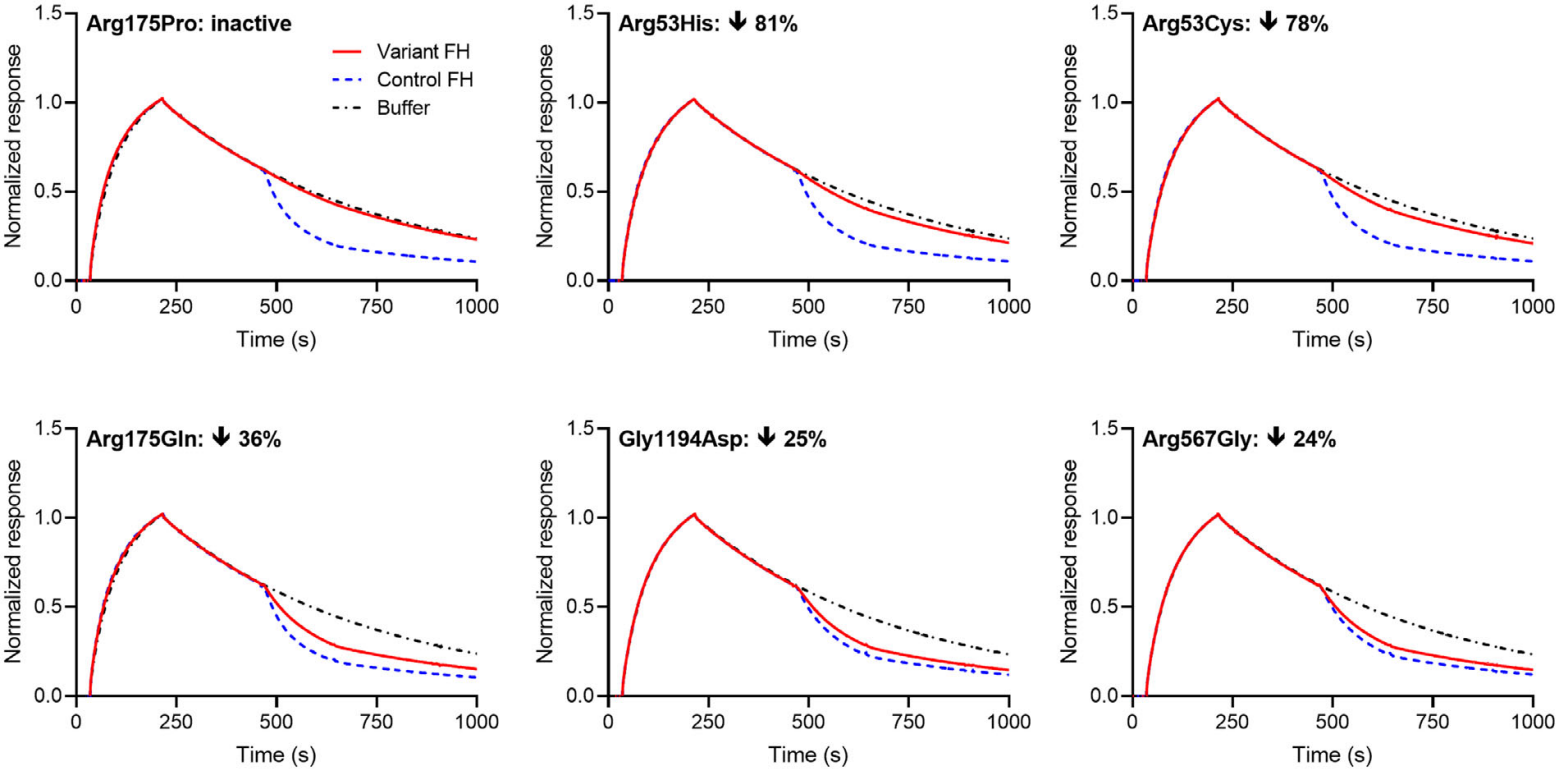
SPR-based measurements of DAA for variants most strongly impacted by their respective substitutions. Each panel contains an overlay of three separately recorded, representative traces for C3bBb assembly and then “decay” (i.e., irreversible dissociation of Bb). One trace (*black*, *dot-dash*) in each panel represents inherent convertase decay; the other two traces were recorded (in sequential experiments, performed in duplicate) in the presence of either control FH (*blue*, *dashed*) or variant FH (*red*, *solid*). The percentage difference between control and variant, in terms of response units observed (normalized to control = 0%) after 175 seconds of FH-mediated C3bBb decay, is indicated within each plot. See also Supplementary Figure S3.

The DAA of FH shortens the operational half-life of the AP C3 convertase and probably requires binding of FH to both its C3b and Bb components.^38^ DAA resides mainly in FH CCPs 1–4 with additional contributions (via C3b binding) from CCPs 19–20.^24^ Time-dependent loss of Bb from C3bBb attached via C3b to an SPR chip was monitored before and after exposure to FH. The resultant traces for variants that appear most impacted by their substitutions—Arg175Pro, Arg53His, Arg53Cys, Arg175Gln, Gly1194Asp, and Arg567Gly—are displayed in Figure 3. Four other variants (Asp130Asn, Arg1210Cys, Arg303Gln, and Pro503Ala) were marginally impacted (Supplementary Fig. S3A). The observed ratios of RUs^variant FH^ to RUs^control FH^, at 175 seconds of decay following FH exposure, ranged from 0.08 to 1.01 for the full set (listed in Supplementary Table S1 and plotted in Supplementary Fig. S3C). A RUs^variant FH^/RUs^control FH^ value (after 175 seconds of decay) of between 0.9 and 1.1 (see Supplementary Table S1 for explanation) was regarded as reflecting insignificant impact, and both Arg2Thr and Leu3Val fell within this range. The remainder of the variants that were close to or indistinguishable from control FH are shown in Supplementary Figure S3B.

#### Impact of AMD-Associated Variations on FI-Mediated CA

**Figure 4.**
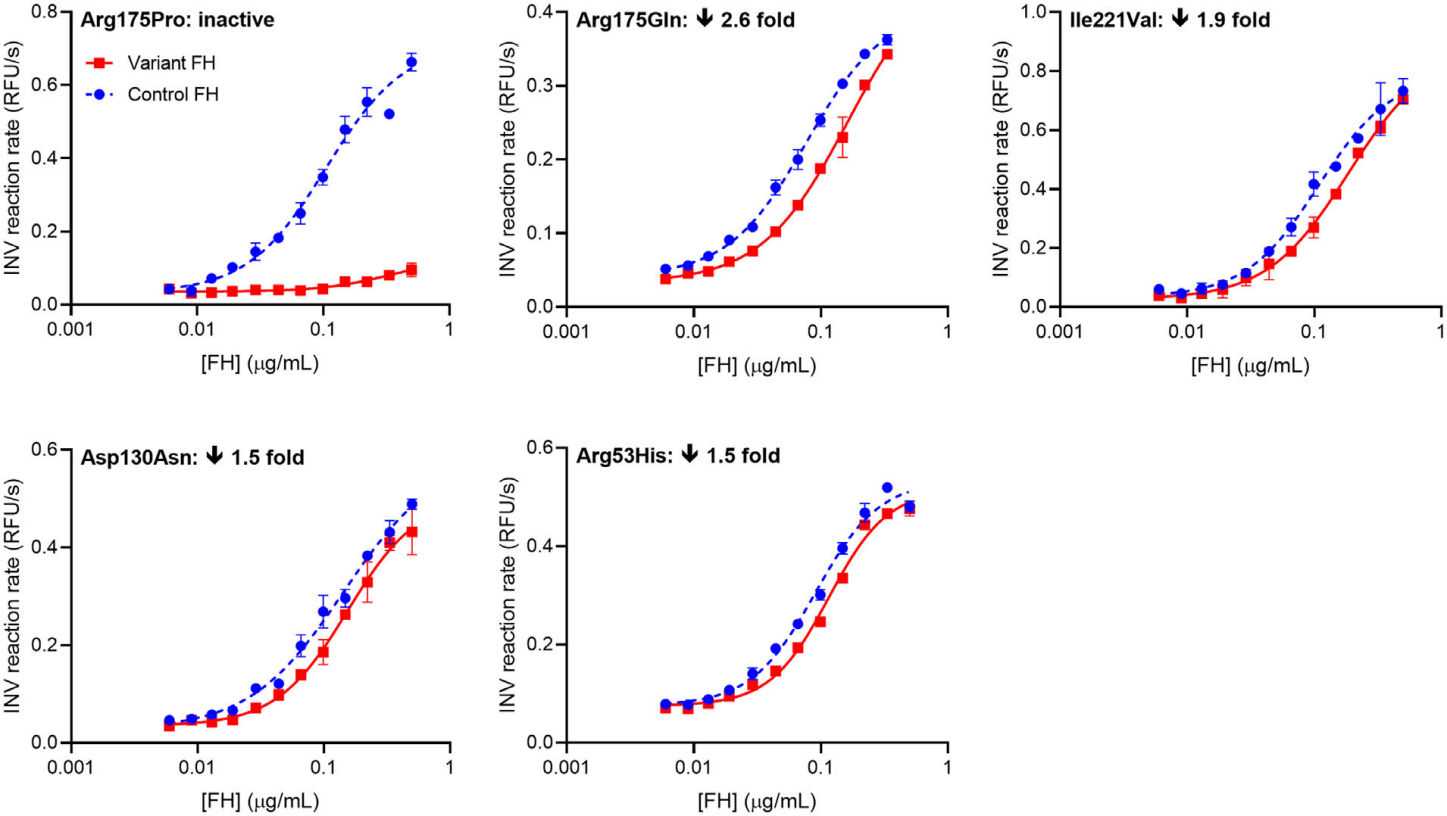
Measurements of CA for the FH variants most strongly impacted by their respective substitutions. Each panel contains overlaid representative plots of rates of change in ANS fluorescence intensity as a function of the concentration (0.03–0.65 µM) of either control (*blue*, *dashed*) or variant (*red*, *solid*) FH added to a mixture of ANS, FI, and C3b (*error bars* correspond to standard deviations for triplicate technical replicates). The increase in fluorescence corresponds to release of bound ANS by C3b as a result of its cleavage by FI. Plots were fitted to generate EC_50_ values. Variant FH and control FH were assayed in duplicate and in a sequential fashion to facilitate a pairwise comparison. The fold-loss in CA (i.e., the fold-increase in EC_50_) for variant versus control is shown within each plot. See also Supplementary Figure S4. INV, inverse.

FH in complex with C3b recruits FI, whereupon FI cleaves C3b to generate iC3b, interrupting the C3b-amplification loop. This ability of FH to exhibit CA for FI was measured in a fluid-phase fluorescence-based assay of C3b integrity.^30^ Multiple EC_50_ values obtained independently for control FH were consistent between replicates within an experiment but varied over a multiple-day measurement series. To reduce experimental variability, we conducted pairwise, same-day comparisons of each FH variant with control FH. Arg175Pro, the most severely affected, and Arg175Gln, Ile221Val, Asp130Asn, and Arg53His comprise the most impacted five variants (Fig. 4). Marginally impacted variants were Arg1210Cys, Gly1194Asp, Gly650Val, Arg303Trp, Arg53Cys, and Ser58Ala (Supplementary Fig. S4A). The activities of the remaining variants appeared unaffected by their substitutions (Supplementary Fig. S4B). Data were also plotted as EC_50_^control FH^/EC_50_^variant FH^ (i.e., values <1.0 imply reduced CA), ranging from 0.07 to 1.13 for the full set (Supplementary Fig. S4C). Values for EC_50_^control FH^/EC_50_^variant FH^ of between 0.8 and 1.2 were regarded as reflecting an insignificant impact (see Supplementary Table S1 for explanation), and both Arg2Thr and Leu3Val fell within this range.

#### Impact of AMD-Associated Variations on FH Performance in an AP Immunoassay

**Figure 5.**
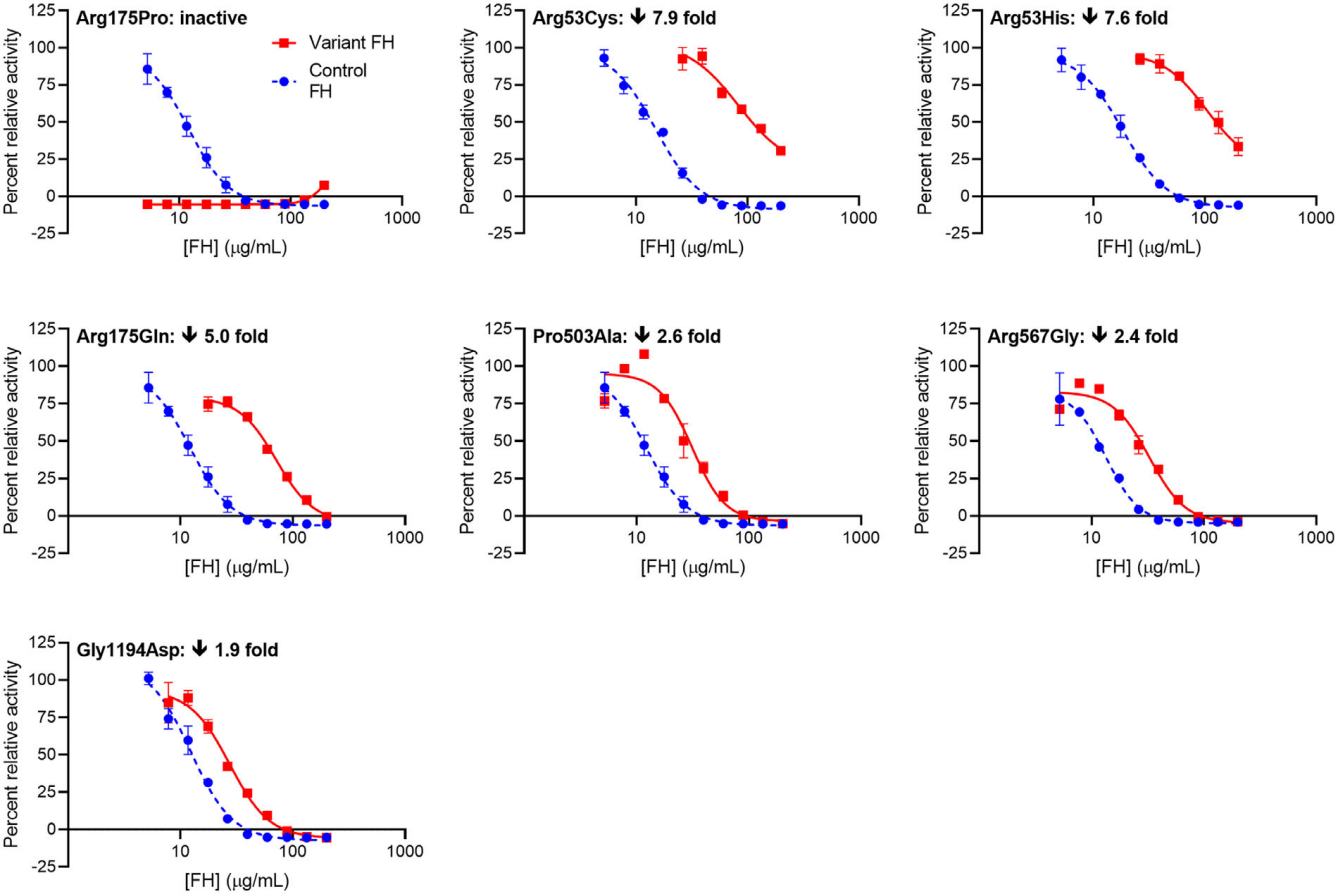
AP immunoassay measurements for the FH variants most strongly impacted by their respective substitutions. Representative data are shown for variants in order of decreasing impact. Each panel contains overlaid plots showing the quantity of sC5b-9 on an AP-activating surface (detected with an antibody to a neo-epitope in the assembled complex) after exposure to FH-depleted serum, as a function of the concentration of added control FH (*blue*, *dashed*) or variant FH (*red*, *solid*) (*error bars* correspond to standard deviations for triplicate technical replicates). Variant and control FH were assayed in a sequential fashion to facilitate pairwise comparisons. Plots were fitted to generate EC_50_ values. The fold-loss in AP-inhibition (i.e., fold-increase in EC_50_) for variant versus control is shown within each plot. See also Supplementary Figure S5.

This immunoassay measures sC5b-9 complexes arising from AP activation in FH-insufficient human serum.^31^ Here, sC5b-9 is generated from complement proteins in human serum under conditions in which the AP is potentially active following addition of Mg^2+^ but the Ca^2+^-dependent classical and lectin pathways remain inactive. Self-amplification of surface-bound C3b may lead to C3b_2_Bb (C5 convertase) formation, generating C5b that can nucleate sC5b-9 assembly. We measured the ability of either control or variant FH to suppress sC5b-9 formation when used to supplement FH-depleted serum. Pairwise (control versus variant) comparisons of proteins tested in this assay exhibited higher experimental variability than observed in more-direct molecular assays. The variants performing most poorly—Arg175Pro, Arg53Cys, Arg53His, Arg175Gln, Pro503Ala, Arg567Gly, and Gly1194Asp—are displayed in Figure 5. Three other variants were classified as marginally affected: Ile221Val, Asp130Asn, and Asp90Gly (Supplementary Fig. S5A), with the remaining appearing unaffected (Supplementary Fig. S5B). Data were also plotted (Supplementary Fig. S5C) as EC_50_^control FH^/EC_50_^variant FH^ (i.e., values <1.0 imply reduced AP suppression), ranging from 0.01 to 1.05 for the full set. EC_50_^control FH^/EC_50_^variant FH^ values between 0.75 and 1.25 were regarded as reflecting insignificant impact (see Supplementary Table S1 for explanation), and both Arg2Thr and Leu3Val fell within this range.

#### Impact of AMD-Associated Variations on FH Performance in a Hemolysis Protection Assay

**Figure 6.**
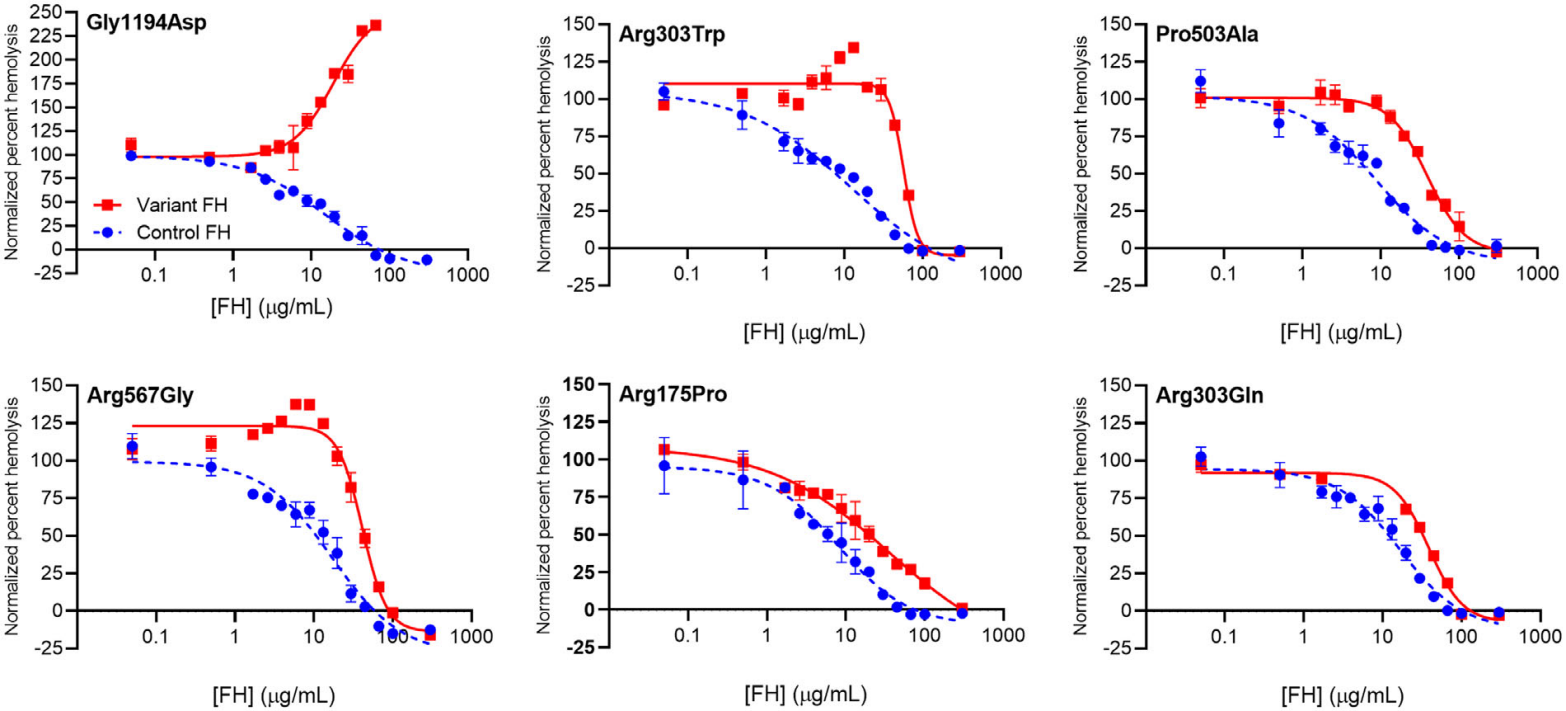
Hemolysis protection measurements for the FH variants most strongly impacted by their respective substitutions. Each panel contains representative overlaid plots of the extent of sheep erythrocyte hemolysis following exposure to FH-deficient normal human serum, as a function of the concentrations of either added control FH (*blue*, *dashed*) or added variant FH (*red*, *solid*) (*error bars* correspond to standard deviations for triplicate technical replicates). Plots were fitted to generate EC_50_ values. Due to reproducibility issues, the derived EC_50_ values were not utilized in calculations of the combined efficacy score; rather, a hemolysis protection score of 0, 0.5, or 1.0 was assigned to severely (as here), marginally, or negligibly affected variants (see Supplementary Fig. S6).

In this assay, SEs were added to FH-depleted serum (in which the AP had been suppressed by storage in EDTA before assay commencement) that had been supplemented with either control or variant FH. The extent of hemolysis was plotted against FH concentration in pairwise, control versus variant, comparisons. SEs are surrogates for human erythrocytes as they bear similar N-glycans and are recognized as “self” by human FH.^39^ Unless exogenous FH is added, both fluid-phase and on-surface AP are activated, with fluid-phase C3b generation being largely futile. Thus, an added FH variant that suppresses fluid-phase AP (preserving C3/FB levels) but is ineffective on self-surfaces might even enhance hemolysis, as could be the case for Gly1194Asp (Fig. 6). No attempt was made to obtain definitive half-maximal inhibitory concentration values using this assay due to variability between experiments performed on different days. Nonetheless, pairwise comparisons were sufficient to indicate which variants are least able to protect SEs. Thus, Gly1194Asp, Arg303Trp, Pro503Ala, Arg567Gly, Arg175Pro, and Arg303Gln emerged as the poorest performers (Fig. 6). Conversely, Arg53His, Arg53Cys, and Arg1210Cys were observed to have a marginal impact on SE hemolysis protection (Supplementary Fig. S6A). Remaining variants had no or minimal observable difference in SE protection from hemolysis compared to control FH (Supplementary Fig. S6B).

### Rank Order of Variants Using a Cumulative Efficacy Score

**Figure 7.**
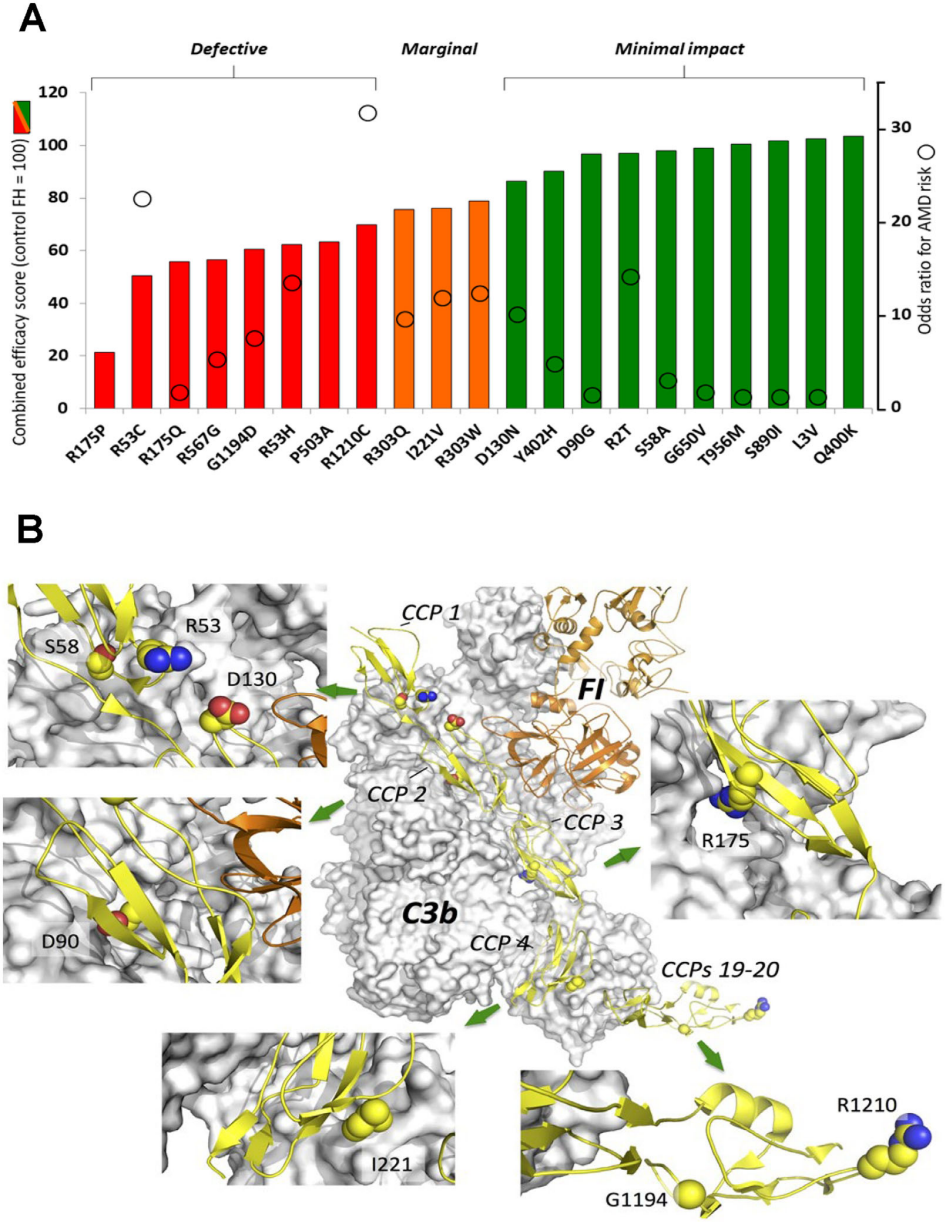
Plots of CES for the variants in this study and their odds ratios (where known) (**A**) and structures (visualized in PyMol) showing details of the locations of substituted residues (**B**). Plot of the CES for each variant in order of increasing CES (that encompasses measurements of both protein quality and quantity; see Materials and Methods); by definition, the CES of control FH = 100. The 10 variants with CES >84% were classified as experiencing “minimal impact,” while 8 variants (shown in *red*) with CES <75% were categorized as “defective.” Three other variants fell between these categories and are regarded as “marginal.” Overlaid on the column plot are the odds ratios, where known (see Table 1), for each variant. In panel **B**, locations are shown, within the atomic structure of the mini-FH:C3b:FI complex (presented in the same orientation as in Fig. 1), of the N-terminal and C-terminal amino acid–residue substitutions among the variants prepared for the current study and discussed in the main text. C3b is represented by a *gray* *surface*, mini-FH by a *yellow cartoon* (with numbered CCP modules), and FI by an *orange cartoon*. The side chains of substituted residues are shown in space-filling representation (*yellow* = carbon, *blue* = nitrogen, and *red* = oxygen) and are labeled in the zoomed-in panels.

For a given FH variant, only weak correlations were observed between the outputs of the various assays when compared with one another (Supplementary Fig. S7). This suggests that an individual in vitro assay may not be strongly predictive of AP-suppressing potential in vivo. Moreover, the in vivo concentration of a variant—both systemically and locally—may also be an important contributing factor. In an attempt to embrace all of the data collected in this study, variants were ranked according to a CES that factored in the level of recombinant protein production as well as the results of all five functional assays (see Materials and Methods), and these scores were plotted (Fig. 7A) along with their AMD-risk odds ratios, and they are represented schematically in a structural context in Figure 7B. A summary of CES data for all variants in the context of their locations within the FH structure is presented in Supplementary Figure S8. Supplementary Figure S9 summarizes the relative contributions of the individual assays to the CES, as well as the impact on the ranking of omitting protein production yields from the CES calculations.

## Discussion

Unlike most previous reports,^18^^,^^39^^,^^40^ the control FH and its 21 single–amino acid residue variants prepared herein are full-length proteins without affinity tags. Despite variability in yield, all were successfully purified in milligram-scale quantities. The amounts of proteins secreted in human cell culture under identical conditions may reflect protein-secretion efficiency in vivo. Hence, the poorer secretion of signal-sequence variant Arg2Thr, compared to Leu3Val and control FH, suggests tissue-specific or systemic haploinsufficiency might contribute to its elevated odds ratio for AMD risk.^41^ Seven of the nine other less-well secreted variants (Arg53Cys, Arg175Pro, Arg175Gln, Ile221Val, Tyr402His, Pro503Ala, Arg567Gly, Gly1194Asp, and Arg1210Cys) might experience folding issues due to an extra cysteine or loss or gain of a structurally consequential proline or glycine or charged residue.

Functional data were reported previously for 17 of the 21 FH variants prioritized for the current investigation, although ours appears to be the first comprehensive study of nontagged, full-length, human cell culture–produced glycoproteins evaluated using assays that interrogate interactions directly at the molecular level where possible. Moreover, we have attempted to rank our FH variants, in terms of their relative impact on complement suppression, by calculating an overall efficacy score that combines, into a single number, the outputs of five functional assays with the measurement of protein secretion discussed above. Merinero et al.^18^ provide an impressive functional audit of >100 aHUS-associated FH variants, including 12 of the AMD-linked variants in the current study. These authors used His-tags for purification, then measured affinity for immobilized C3b in an enzyme-linked immunosorbent assay. They assessed AP regulation by comparing protection of guinea pig erythrocytes from hemolysis in FH-depleted human serum. To compare CA, the authors monitored sensitivity to hemolysis in rat serum of human C3b-decorated SE after human FI treatment in the presence of each FH variant. To assess DAA, they compared the effects of incubation with each variant on the hemolytic susceptibility of C3bBb-decorated SE. In their assays, Arg2Thr, Gln400Lys, Tyr402His, Gly650Val, and Thr956Met behaved like control FH, whereas Arg53Cys, Arg175Pro, Gly1194Asp, and Arg1210Cys were classified as pathogenic.

These results are largely in agreement with our findings. However, while we categorized Ile221Val and Arg303Gln as “marginal” and Pro503Ala as “severely affected,” Merinero et al.^18^ detected no activity loss for these or other substitutions located more centrally within the FH molecule. Ile221Val conservatively replaces a CCP 4 residue that contacts C3b but is not entirely buried at the intermolecular interface (Fig. 7B). Ile221Val owes its “marginal” classification to poorer CA and AP control. Remote from the FI-binding site, Ile221 could interact with CCP 18 in the FH:C3b complex. Arg303Gln emerged as “marginal” because of its poorer C3b binding and lower protection of SEs. Exposed on CCP 5, Arg303 could interact with C3b or the C-terminal CCPs of FH in the FH:C3b complex. Pro503Ala—replacing a proline buried near the C-terminal pole of CCP 8, close to CCP 9 (Fig. 1, Fig. 7B)—was a poor C3b binder and performer in our AP immunoassay and defective in SE hemolysis protection. Yet we observed only a marginal effect on DAA of Pro503Ala and a negligible effect on CA. Thus, P503A is an outlier in plots that attempt to correlate the results of the various assays with one another (Supplementary Fig. S7). This, as well as the discrepancies between our and previous reports, suggests that consequences of substitutions that affect the conformation of the FH molecule, rather than directly impacting on its binding sites, may be more easily overlooked. They could nonetheless be important in terms of an overarching AP-suppressing role of FH in vivo.

Five further variants studied herein were also investigated in other previous studies. Three reports agreed with our findings: Arg53His in a full-length *Pichia* *pastoris*–produced version of FH was severely compromised,^42^ Asp130Asn in an affinity-tagged FH 1 to 4 construct had only modest functional effects,^43^ and Ser890Ile in the context of FH purified from patient plasma was fully functional.^44^ A report for a fourth variant, Ser58Ala purified from patient plasma, found defects in hemolysis protection and CA and DAA on erythrocytes,^45^ whereas our Ser58Ala FH was impacted solely in terms of CA. Ser58 is not surface exposed, but a change to Ala could induce local structural or dynamic perturbations in a C-terminal loop of CCP 1 that is part of the C3b-binding surface. Finally, Asp90Gly in an affinity-tagged FH 1 to 4 construct had low CA on a C3b-decorated SE,^46^ whereas we detected no impact on fluid-phase CA. While Asp90 is located at the CCP 2:C3b interface (Fig. 1, Fig. 7B), its substitution to Gly had no measurable effect on C3b binding, or on DAA, in either the FH 1 to 4 context or in our own studies.

Of the previously unstudied variants in our set, Leu3Val, in the signal sequence, was secreted at good yield (unlike Arg2Thr) and displayed full activity in our assays. Arg175Gln was categorized as functionally impaired. Although it was less impacted than Arg175Pro, this adds evidence for Arg175, which is located at the CCP 3:C3b interface, being critical for FH function. Arg303Trp was impacted to a similar extent as Arg303Gln (both categorized as “marginal”), reinforcing the finding that CCP 5 might be considered as part of the “N-terminal” C3b-engaging region of FH. Finally, Arg567Gly was categorized as functionally impactful in the current study. Arg567 lies in the CCP 9 to 10 junction (Fig. 1, Fig. 7B).

Our data for the previously unexplored Arg567Gly variant, in conjunction with our new data for Pro503Ala, emphasize that central CCP modules are not functionally irrelevant and hence that sequence variations here can potentially disrupt AP suppression by FH and contribute to AMD. A bacterial protein that hijacks FH by binding to CCPs 9 and 10 was previously shown to augment FH function.^39^ These observations support the functional and architectural importance for FH of its central region,^34^^,^^35^ reinforcing the concept that FH operates as a coherent whole rather than the sum of its several C3b-binding and surface-recognition sites. Thus, a strategy whereby full-length FH is administered therapeutically could have advantages over the therapeutic use of engineered “mini” versions of FH.^47^^–^^50^

In this study, we summed (without differential weightings) normalized data from all five assays of FH's canonical complement-regulating functions along with the protein yield and divided by 6 (and multiplied by 100) to calculate for each variant a CES, with respect to AP suppression, whereby control FH has a CES of 100. Note that because the hemolysis protection assay displayed more experimental variability than other assays, we used a ternary score (1 = protection similar to that of control FH; 0 = protection consistently compromised; 0.5 = in between) in the CES calculations. The CES likely reflects both the quantity of an FH variant and its quality (functional competence) as a regulator of the AP. Hence, it could indicate the likelihood that the possession of a specific *CFH* variant will have an impact on the proper operation of an individual's complement system. It might be argued that the protein-yield component of the CES is less reliable than the other components since numbers are based on a single protein production run, and they may not, in any case, reflect levels of protein secreted in vivo. As shown in Supplementary Figure S9, omission of protein yield from the calculation of CES has little impact on the rank order of variants.

By definition, “nonrisk” (control) FH has an AP-regulatory CES of 100. Minimally impacted are Arg2Thr, Leu3Val, Ser58Ala, Asp90Gly, Asp130Asn, Gln400Lys, Tyr402His, Gly650Val, Ser890Ile, and Thr965Met, which have CESs between 86 and 104. Nevertheless, of these, Arg2Thr showed reduced expression and, therefore, could be considered a “risk” variant on this basis alone. On the margin of this group are Ile221Val, Arg303Gln, and Arg303Trp. If the primary goal were to correct only for complement dysregulation, our data might indicate insufficient support for prioritization of individuals having these variants for clinical trials of FH administration, although our study should be weighed against others that reported functional abnormalities for Ser58Ala and Asp90Gly (see above). Moreover, even marginally or minimally impacted (from an AP-regulatory perspective) variants may potentially contribute significantly to pathogenicity of AMD, considering the disease is age related and arises as a result of cumulative tissue damage suffered progressively over several years. Eight variants have an AP-regulatory CES below 75, a cutoff below which we have defined variants as being “defective.” In order of decreasing CES, the variants of interest are Arg1210Cys, Pro503Ala, Arg53His, Gly1194Asp, Arg576Gly, Arg175Gln, Arg53Cys, and Arg175Pro. Note that if protein yields are omitted from the CES (Supplementary Fig. S9), Arg1210Cys moves out of the bottom eight places in the ranking and is replaced by Arg303Gln. Overall, based on the data presented here, it seems unlikely that complement would be adequately regulated in patients carrying variants with low CES values. Indeed, a prior study showed that the rare Arg175Pro variant, which had the lowest CES, is associated with early-onset advanced AMD.^8^ Hence, our data argue for therapeutic administration of control FH to these individuals as a way to correct complement regulatory deficiency.

Although the present study fulfills the objectives of helping to narrow a patient pool for targeted treatment of AMD with FH-based therapeutics, we acknowledge that more biological replicates would strengthen our conclusions. Furthermore, the correlation between odds ratios for AMD risk and CES values (Fig. 7A) is weak, suggesting that roles of FH other than AP regulation should also be explored as contributors to AMD pathogenesis and progression.^27^^,^^51^ Its roles in managing dysregulation of lipid metabolism and retinal pigment epithelium oxidative stress, for example, have not been evaluated in this study. This reinforces the importance of augmenting our analysis with additional assays that interrogate noncomplement, noncanonical pathways.

In conclusion, we applied a panel of complement-based in vitro assays to a set of recombinantly produced, full-length, FH variants that are putatively linked to an enhanced risk of developing AMD. In general, a good agreement with previous measurements, where available, validated our approach. We were able to categorize (using a combined efficacy score) the AP-regulatory competence of >20 variants and identify previously unrecognized, potentially pathogenic, functional impairments arising from substitutions in the central modules of FH. This approach could be expanded to other variants, including those of unknown functional significance. It should be combined with other assays that assess noncanonical FH functions, aiming to facilitate the selection of cohorts that are most likely to benefit from therapeutic administration of FH.

## Supplementary Material

Supplement 1
