# Supplementary material for: An Evaluation of the Complement-Regulating Activities of Human Complement Factor H (FH) Variants Associated With Age-Related Macular Degeneration
Source: Invest Ophthalmol Vis Sci. 2022 Nov 29;63(12):30. doi: 10.1167/iovs.63.12.30 (PMC9716232; doi:10.1167/iovs.63.12.30)
Supplement: Supplement 1 [file iovs-63-12-30_s001.pdf]

Supplement

Supplementary Methods

Supplementary Methods I: Expression and purification of recombinant FH variants

Samples of the following prioritized 21 FH variant proteins associated with enhanced AMD risk, and a control FH protein (ID P08603 in UniProtKB), were prepared:

Arg2Thr*	Arg303Gln
Leu3Val*	Gln400Lys
Arg53Cys	Tyr402His
Arg53His	Pro503Ala
Ser58Ala	Arg567Gly
Asp90Gly	Gly650Val
Asp130Asn	Ser890Ile
Arg175Gln	Thr956Met
Arg175Pro	Gly1194Asp
Ile221Val	Arg1210Cys
Arg303Trp	

* Amino-acid substitution in the N-terminal signal sequence, which is cleaved from mature, secreted protein

All proteins were produced (without affinity tags) by transient expression using a standard method in human cell culture (HEK293 cell lines),[1] secreted (with cleavage of N-terminal signal sequence, residues 1-18), and purified to homogeneity (Supplementary Fig. 1A) from conditioned media using a proprietary affinity-purification method. Control FH and all FH variants were produced and purified in an identical manner; yields of purified proteins were determined by measuring absorbance of light at 280 nm in a Nanodrop (Thermo Fisher Scientific, Waltham, MA, USA) and applying calculated extinction coefficients.

Supplementary Methods II: C3b binding by surface plasmon resonance (SPR)

For each experiment, 0.25 µM variant FH (or control FH) in 10 mM 4-(2-hydroxyethyl)-1-piperazineethanesulfonic acid (HEPES) buffer, 150 nM NaCl, 0.05% (v/v) surfactant P20, was injected in duplicate over amine-coupled C3b (300 RUs on a C1 chip) for 150 seconds at 50 µL/minute followed by a dissociation time of 600 seconds. The sensor chip surface was regenerated between individual sample injections by three 30-second 50 µL/minute injections of 1 M NaCl, with a surface stabilization period of 150 seconds following the third NaCl injection. Injections of control FH – for pairwise comparisons with each variant FH - were replicated at frequent, regular intervals to help take account of any drift in chip or instrument performance over the duration of the experiment. The baseline drift was corrected by subtracting the signal obtained from an injection of 0 µM FH. Data were analyzed using the Biacore Evaluation software and a 1:1 steady-state binding model.

Supplementary Methods III: Decay accelerating activity (DAA) assay

The SPR experiments were performed using 10 mM HEPES, 150 mM NaCl, 0.05% (v/v) surfactant P20, 1 mM $MgCl_2$ (pH 7.4) (GE Healthcare). C3bBb was assembled on the CM5 chip (GE Healthcare) bearing 2845 RUs of immobilized (amine-coupled) C3b molecules by performing a 220-second 10 µL/minute injection of a solution containing 500 nM FB (Complement Technology, Inc.) and 50 nM FD (Complement Technology, Inc.). The decay of C3bBb was subsequently monitored over an initial 230-second dissociation phase in the absence of any FH, allowing observation of intrinsic convertase decay (*i.e.*, a decline in RUs attributable to loss of Bb). Subsequently, a solution of 20 nM FH (or the control) was injected at 10 µL/minute for 550 seconds, allowing observation of accelerated dissociation. The surface was regenerated between measurements by a 30-second 10 µL/minute injection of 0.1 µM plasma-purified FH followed by three, 30-second, 10 µL/minute injections of 1 M NaCl, and then a surface-stabilization period of 120 seconds. The SPR response arising from the binding of variant or control FH to immobilized C3b, in the absence of the convertase, was subtracted from the corresponding convertase decay response. Data were processed using the Biacore Evaluation software and then normalized to compensate for the small drift in signal (assumed to arise from the gradual leaching of C3b from the surface over multiple measurements). Normalization of data was achieved by comparing responses at 220 seconds (*i.e.*, at the time when the injection of FB and FD ceased) and adjusting these to be 1.00. The normalized responses were then plotted against time, and the resultant plots overlaid at the time point of variant/control FH injection.

Supplementary Methods IV: Fluorescent cofactor assay

Stock solutions of variant FH and control FH were serially diluted in Tris-buffered saline (1X TBS; 50 mM Tris, 150 mM NaCl, pH 7.4) to achieve concentrations ranging from 0.025 to 0.5 mg/mL. Subsequently, assay components were added to opaque half-area black polystyrene plates in the following order in a 50 µL final reaction volume: 0.02 mg purified human C3b (Complement Technology, Inc.), 5 µM ANS (final concentration) (Acros Organics B.V.B.A., Geel, Belgium), control/variant FH to give final concentrations between 5 and 100 µg/mL, and 0.1 µg of FI (Complement Technology, Inc.). Reactions were mixed briefly by shaking at 4000 revolutions per minute and monitored over 30 minutes at 30-second intervals at 30 °C. Fluorescence readings were recorded in kinetic mode with excitation set to 386 nm and emission set to 472 nm.

Supplementary Methods V: Hemolysis assay

Human FH-depleted serum (20 µL) (Complement technology, Inc.) was preincubated at room temperature for ten minutes with either variant FH or control FH at concentrations ranging from 25 to 600 µg/mL using gelatin-containing veronal buffer (GVB^0 Complement Technology, Inc.) with 0.1 mM EDTA [$GVB^{0.1\ mM\ E}$], as the diluent. Sheep erythrocytes (Complement Technology, Inc.) were washed once in GVB^0 and re-suspended to $2.1x10^8$ cells/mL in $GVB^{0.1\ mM\ E}$. A total of $2.1x10^7$ sheep erythrocytes were added to each well of a 96-well plate followed by Mg·EGTA at a

final concentration of 10 mM in a total reaction volume of 200 µL. Wells containing reaction mixes in heat-inactivated FH-depleted serum and 1% v/v triton-X (BioVision, Milpitas, CA, United States) in water were included as negative and positive controls for lysis, respectively. Samples were incubated at 37 °C for one hour with shaking, followed by the addition of 150 µL of $GVB^{10\ mM\ E}$ to stop further cell lysis. The samples were centrifuged at 1000 x g for five minutes at 7 °C. A volume of 150 µL of pre-cleared supernatant was transferred to a clear-bottom 96-well plate. The extent of hemolysis (hemoglobin released) was then measured, in triplicate samples for each FH concentration, as absorbance at 412 nm, corrected for background absorbance measured at 690 nm.

Supplementary Tables

Supplementary Table 1. *FH variants data summary.* Table showing an overview of the results of the 21 FH variants in the five functional assays.

VARIANT	Protein Expres-sion	C3b Binding	Cofactor Activity	Decay Accele-ration Activity	AP Immuno-assay	Hemo-lysis	CES	Functio-nally Impaired?
Control CFH Normal Range →	83 mg	0.9-1.2*	0.8-1.2*	0.9-1.1*	0.75-1.25*	1	100	NO
Arg2Thr	0.72	1.21	1.13	1.01	0.75	1	97.00	NO
Leu3Val	0.96	1.08	1.10	1.01	1.00	1	102.50	NO
Arg53Cys	0.54	0.87	0.77	0.22	0.13	0.5	50.50	YES
Arg53His	1.30	0.97	0.65	0.19	0.13	0.5	62.33	YES
Ser58Ala	1.20	0.98	0.77	0.95	0.98	1	98.00	NO
Asp90Gly	1.00	1.21	0.97	0.95	0.68	1	96.83	NO
Asp130Asn	1.07	0.98	0.67	0.83	0.63	1	86.33	NO
Arg175Gln	0.35	0.78	0.38	0.64	0.20	1	55.83	YES
Arg175Pro	0.26	0.86	0.07	0.08	0.01	0	21.33	YES
Ile221Val	0.49	0.99	0.53	0.96	0.59	1	76.00	Marginal
Arg303Trp	1.09	1.10	0.76	0.96	0.82	0	78.83	Marginal
Arg303Gln	1.16	0.83	0.83	0.88	0.84	0	75.67	Marginal
Gln400Lys	1.24	1.00	0.93	0.99	1.05	1	103.50	NO
Tyr402His	0.74	1.00	0.88	1.00	0.79	1	90.17	NO
Pro503Ala	0.66	0.84	1.07	0.85	0.38	0	63.33	YES
Arg567Gly	0.76	0.65	0.81	0.76	0.42	0	56.67	YES
Gly650Val	1.22	1.02	0.75	0.99	0.96	1	99.00	NO
Ser890Ile	1.02	1.06	0.97	1.01	1.05	1	101.83	NO
Tyr956Met	1.18	0.97	1.11	0.94	0.83	1	100.50	NO
Gly1194Asp	0.35	1.27	0.75	0.75	0.51	0	60.50	YES
Arg1210Cys	0.43	0.89	0.73	0.88	0.76	0.5	69.83	YES

*Average measurements were calculated for the multiple 21 individual control FH runs and then comparison of the individual control FH runs to the average provided a standard deviation that reflected experimental error. This was used to infer the range of “normal” (non-impacted by the substitution), which is an upper estimate since it includes the variation in control FH results over the entire duration of the measurement series and does not take into account the sequential nature of the pairwise measurements used to calculate the ratios).

Abbreviations: AP, alternative pathway; C3b; complement control protein 3b; CES, combine efficacy score; FH, complement factor H

Supplementary Table 2. *Summary of K_D measurements for control FH and a selection of variants.*

	K_D (µM)	±SE†	R_{max} (RU)	χ^2 (RU)	Offset (RU)
**Control FH***	1.70	0.08	481	5.12	4.74
**Arg53His**	3.16	0.1	460	0.6	1.97
**Arg175Pro**	6.39‡	2.1	770	24.7	-6
**Arg567Gly**	4.21	0.12	578	0.37	1.33
**Arg1210Cys**	1.95	0.11	436	4.95	4.3

* In SPR experiments, each sample was injected at a range of concentrations (0.0156–2 µM), in duplicate, over 1500 RUs of C3b that had been amine coupled on a Biacore CM5 sensor chip.

† The K_D ± SE is for the fit of the data to a 1:1 equilibrium model using Biacore software (that was also used to generate the values of R_{max}, χ^2 and offset).

‡ Unreliable value: SPR profiles featured a low RU gain during injection, and a slow return to baseline; there was a poor fit to the 1:1 binding model.

Abbreviations: K_D, equilibrium dissociation constant; R_{max}, maximum binding response; RU, response units; SE, standard error; SPR, surface plasmon resonance

Supplementary Figures

Supplementary Figure 1A. *Representative SDS-PAGE gels to assess purity of the FH variant samples.* In each lane, 10 µL of a 2.5 µM stock solution of each variant (later used for SPR experiments to measure C3b binding) was denatured in SDS, heated at 100 °C for five minutes and run, as shown in the key, under either non-reducing or reducing (500 mM dithiothreitol (DTT)) conditions. The results for nine representative variants are shown. Of the 22 proteins prepared for this study (including 13 not shown), only Gly1194Asp showed evidence of a contaminating, dimer band (that disappeared under reducing conditions).

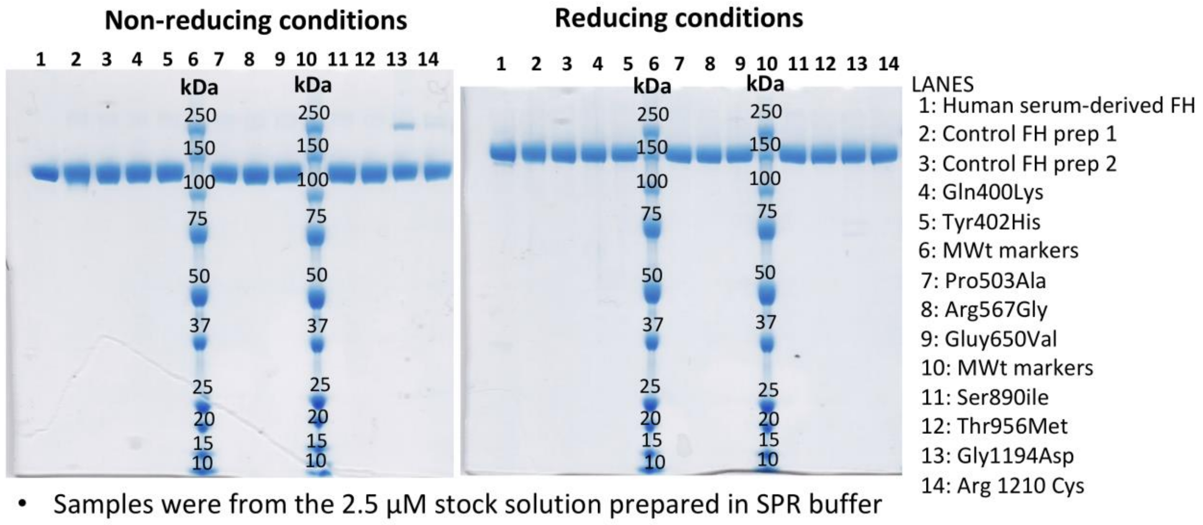

Abbreviations: C3b; complement control protein 3b; FH, complement factor H; MWt, molecular weight; SDS-PAGE, sodium dodecyl-sulfate polyacrylamide gel electrophoresis; SPR, surface plasmon resonance

Supplementary Figure 1B. *Yields of protein.* The quantity of secreted protein obtained after affinity purification was estimated by absorbance of light at 280 nm and normalized (at 1.00) to the yield of purified control FH (83 mg from one liter of cell culture). All of the protein-production runs and FH purifications were carried out once, using identical procedures. Red bars indicate below-average yield.

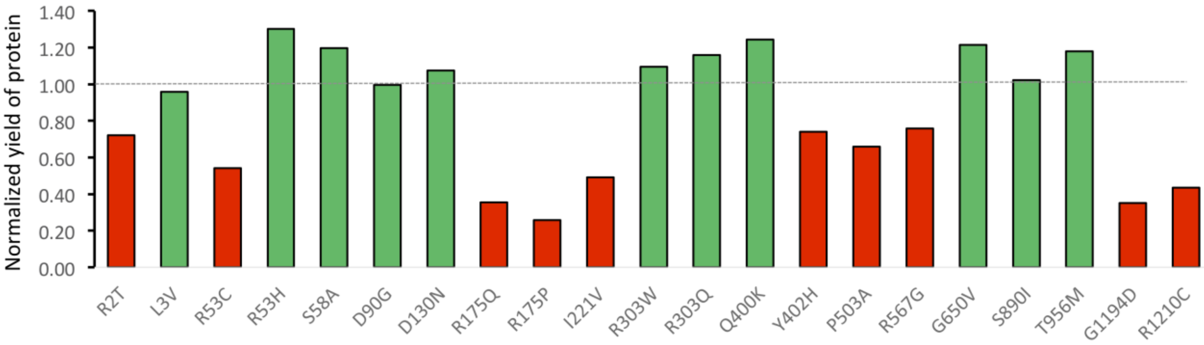

Abbreviations: FH, complement factor H

Supplementary Figure 2A. *Unusual C3b-binding SPR trace for Gly1194Asp.* Representative measurement of Gly1194Asp (red) and control FH (blue) binding to amine-coupled C3b on a C1 chip (performed in duplicate). Unlike any of the other 20 FH variants in the current study, Gly1194Asp binds more rapidly and produces a greater response than control FH.

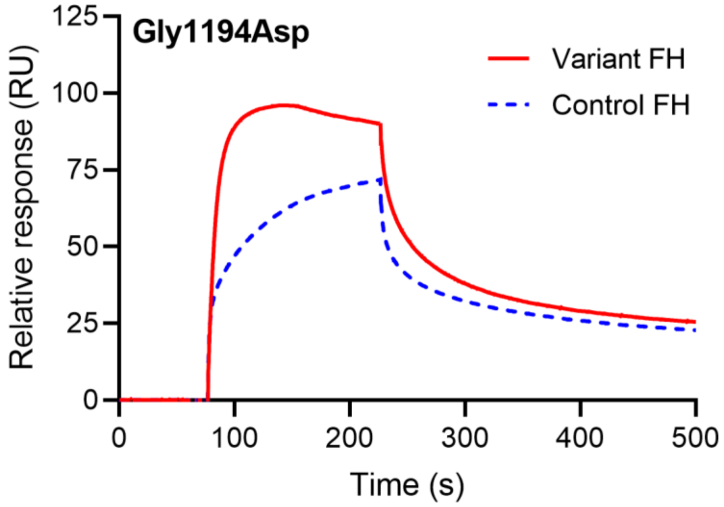

Abbreviations: C3b, complement component 3b; FH, complement factor H; RU, response units; s, seconds; SPR, surface-plasmon resonance

Supplementary Figure 2B. *SPR traces for FH variants that bind to immobilized C3b to an extent that is not severely impacted by their respective substitutions.* In each panel, representative traces obtained for variant and control FH, in a pairwise comparison (performed in duplicate), are overlaid. The panels are arranged in order of increasing residue number.

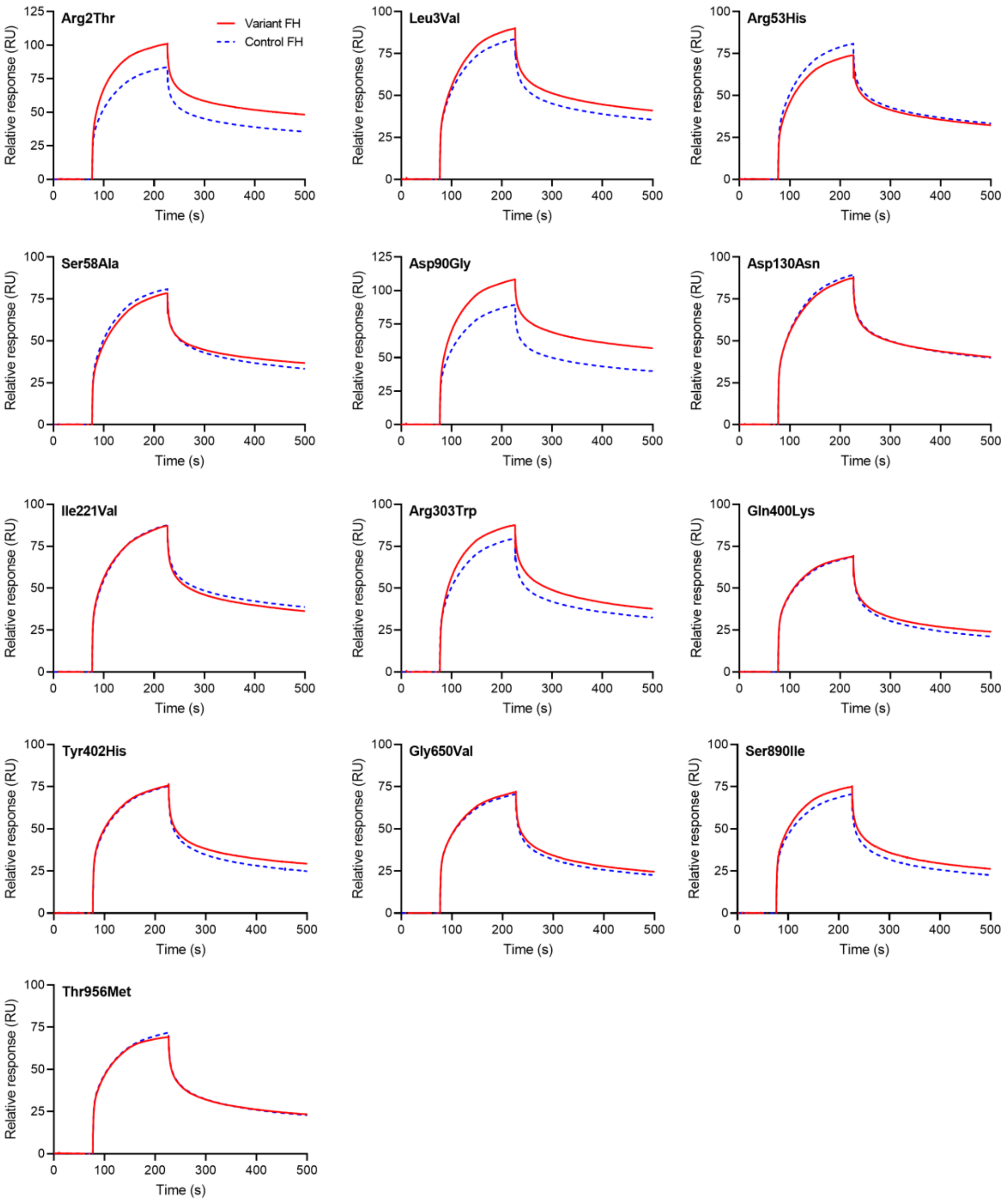

Abbreviations: C3b, complement component 3b; FH, complement factor H; RU, response units; s, seconds; SPR, surface plasmon resonance

Supplementary Figure 2C. *Summary of C3b binding by FH variants.* A column plot showing the $RU^{variant\ FH}/RU^{control\ FH}$ ratio, as measured by SPR at 220 seconds (binding equilibrium), for each variant in order of increasing residue number. The dotted lines correspond to the “normal” range (see Supplementary Table 1). The seven poorest C3b binders (selected for display in Fig. 2 in the main paper) are highlighted in red.

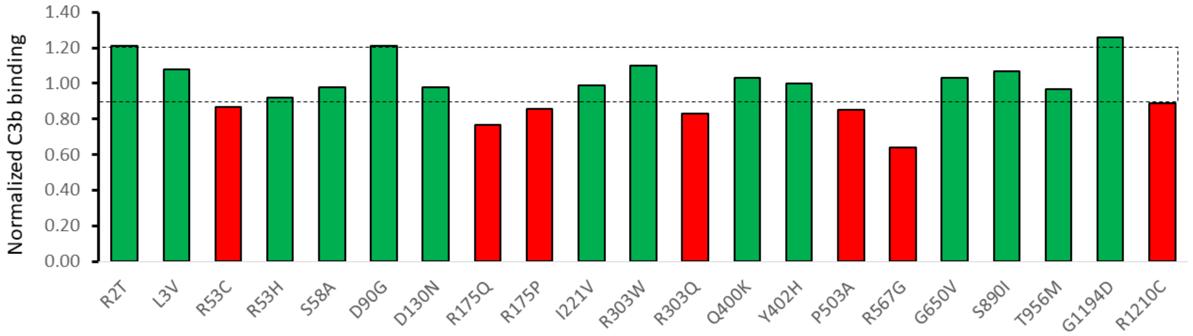

Abbreviations: C3b, complement component 3b; FH, complement factor H; RU, response units; SPR, surface-plasmon resonance

Supplementary Figures 3A and B. *SPR-based measurements of decay-accelerating activity (DAA) for variants not severely impacted by their respective substitutions.* Data for (A) marginally impacted FH variants, or (B) minimally impacted FH variants, are shown. Each panel contains representative (of duplicate) traces for the formation of C3bBb and its subsequent "decay". One trace (black, dotdash) in each panel shows the formation and the natural decay of the convertase. The other two traces, recorded in a pairwise fashion, show the potential acceleration of convertase decay upon injection over the chip of either control FH (blue, dashed) or variant FH (red, solid). In panel A, the percentage difference between control and variant, in terms of response units observed (normalized to control = 0%) after 175 seconds of accelerated C3bBb decay, is indicated within each plot.

A

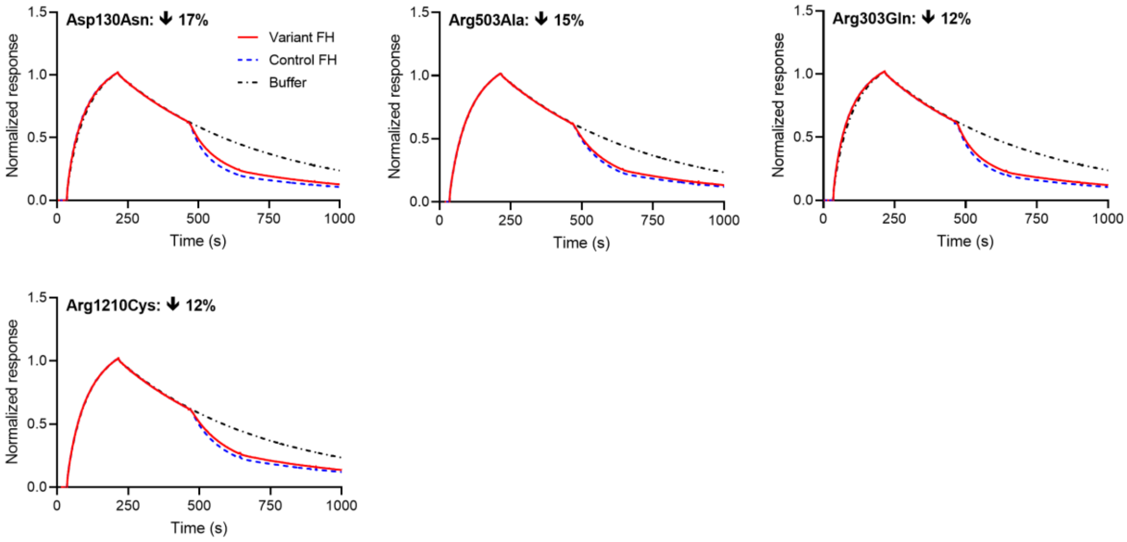

B

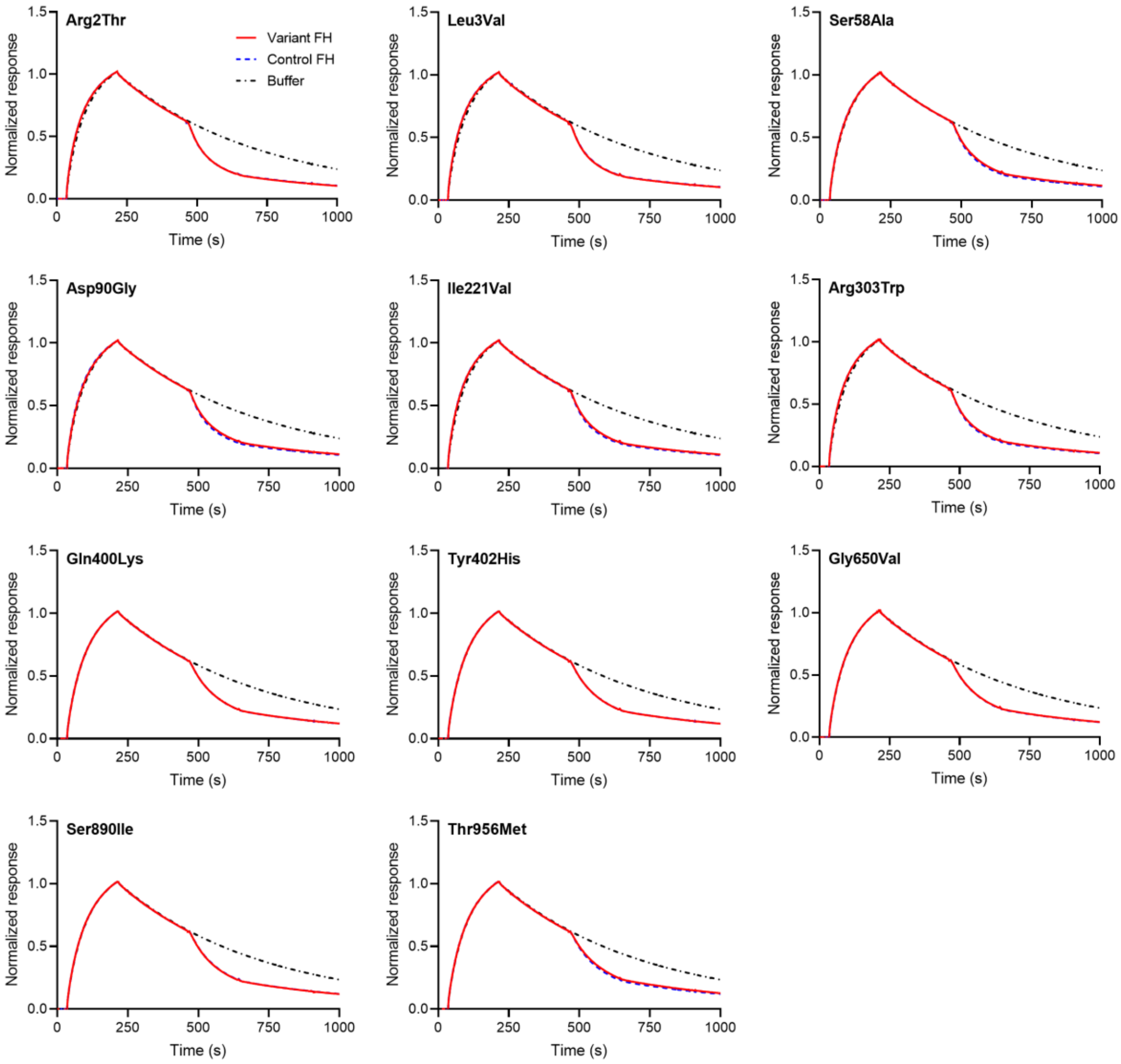

Abbreviations: C3b, complement component 3b; C3bBb, C3 convertase; FH, complement factor H; RU, response units; s, seconds; SPR, surface-plasmon resonance

Supplementary Figure 3C. *Summary of DAA measurements for FH variants.* A column plot showing the ratios of ($RU^{variant\ FH}$)/($RU^{control\ FH}$), after 175 seconds of decay, for each FH variant in order of increasing residue number. The dotted lines correspond to the “normal” range (see Supplementary Table 1). The six poorest decay accelerators (selected for display in Fig. 3 in the main paper) are highlighted in red.

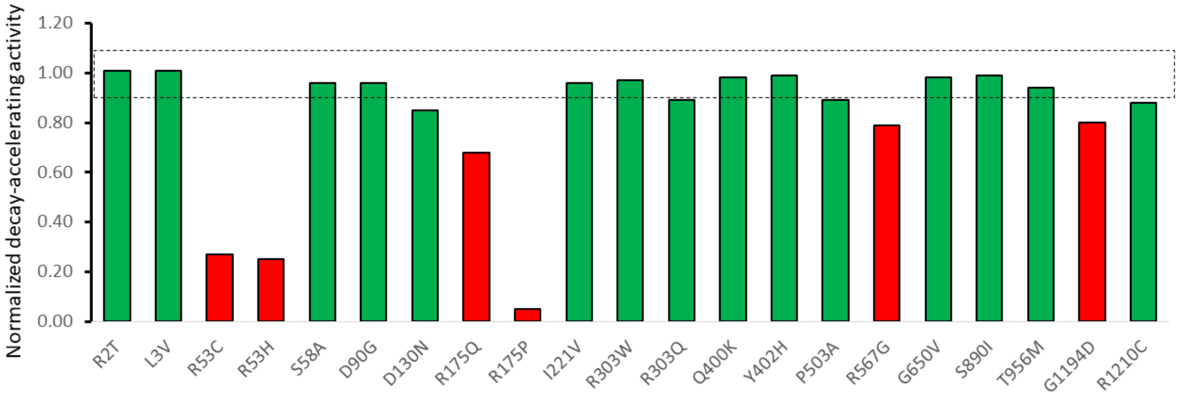

Abbreviations: DAA, decay accelerating activity; FH, complement factor H; RU, response units

Supplementary Figures 4A and B. *Cofactor activity measurement for variants not severely impacted by their respective substitutions.* Representative data for (A) marginally impacted FH variants, or (B) negligibly impacted FH variants. Each panel contains overlaid plots of the rate of change in ANS fluorescence intensity (upon incubation of 2.27 µM C3b with 22.73 nM FI) as a function of the concentration (0.03 - 0.65 µM) of either control or variant FH (error bars correspond to standard deviations for triplicate technical replicates). The change in fluorescence signal corresponds to the release of bound ANS by C3b as a result of its cleavage by FI, and plots were fitted to generate EC_{50} values. In panel A, the fold difference in EC_{50} value, of variant with respect to control FH, is indicated for each variant.

A

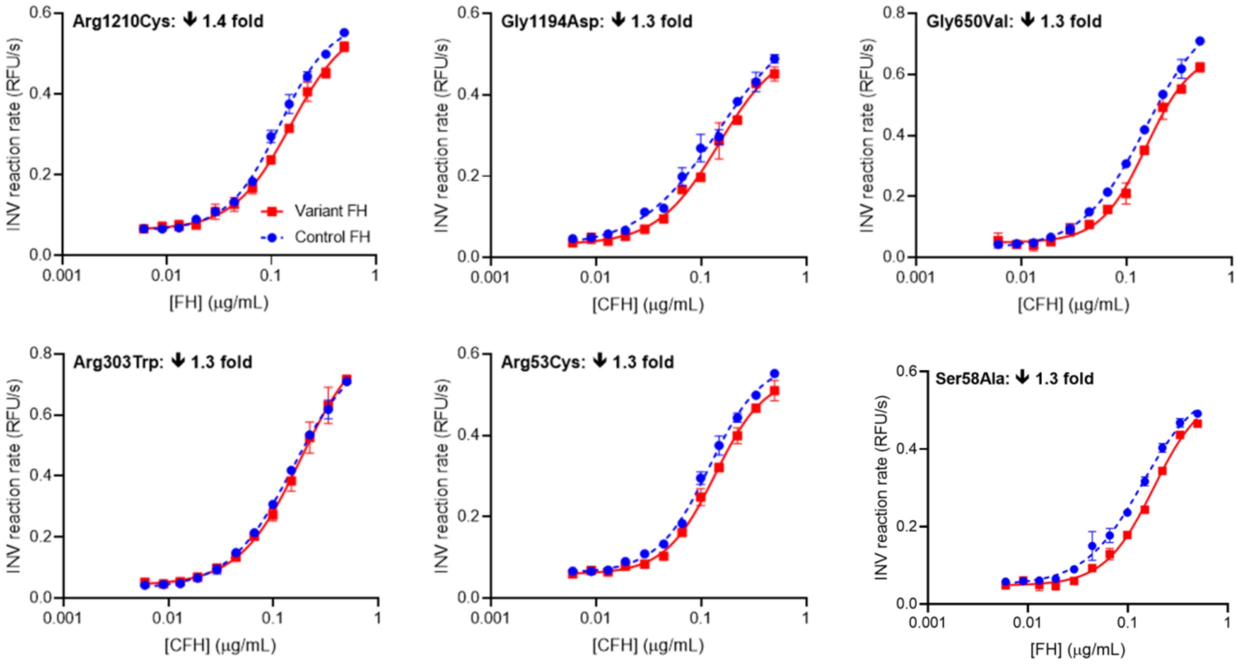

B

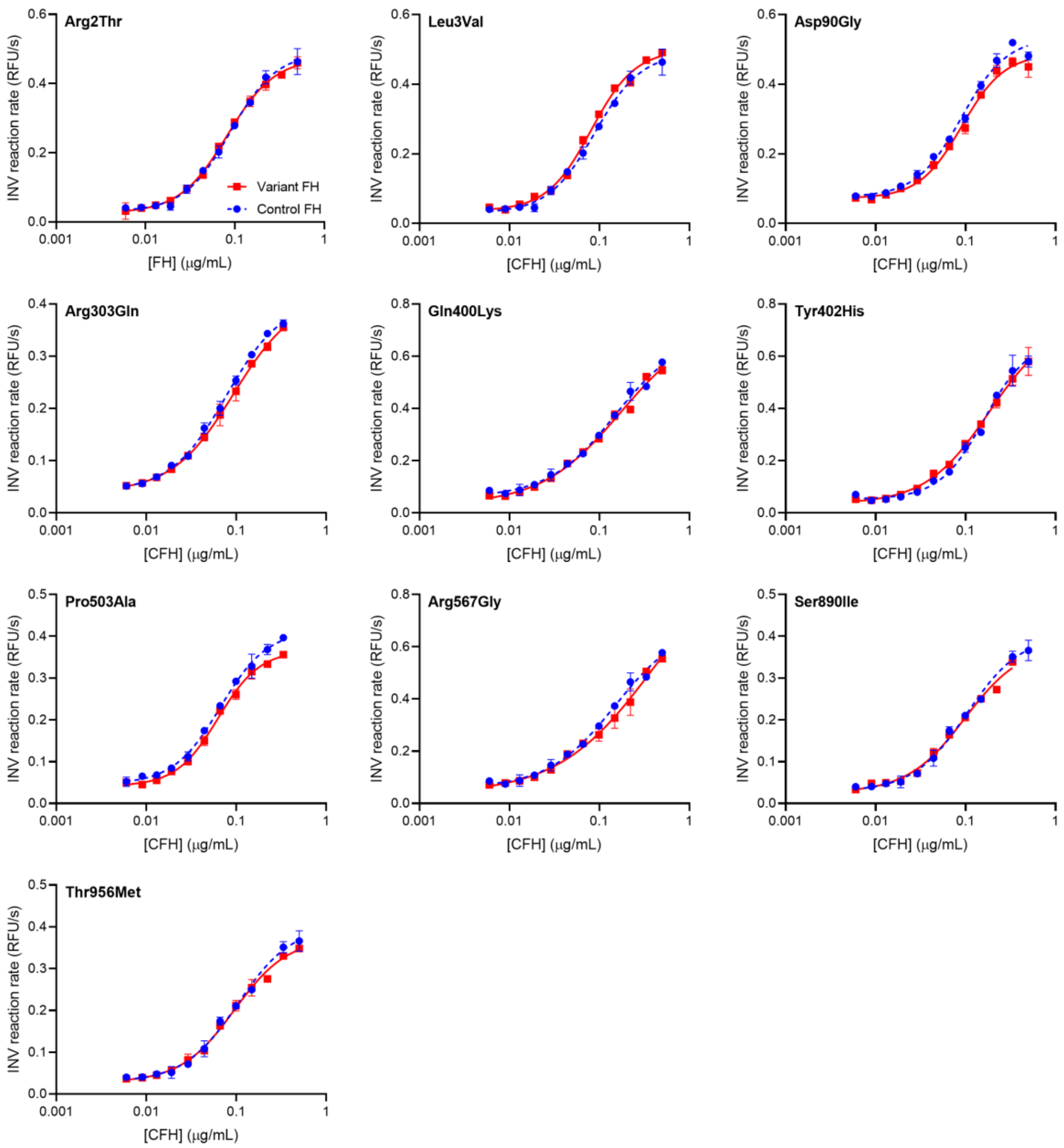

Abbreviations: ANS, 8-anilinonaphthalene-1-sulfonic acid; C3b, complement component 3b; CA, cofactor activity; EC_{50}, half-maximal effective concentration; FH, complement factor H; FI, complement factor I; INV, inverse; RFU, relative fluorescence units; s, seconds

Supplementary Figure 4C. *Summary of CA measurements for FH variants.* A column plot showing the ratios of $EC_{50}^{\text{control FH}}$/ $EC_{50}^{\text{variant FH}}$ (*i.e.*, values <1.0 imply reduced CA) for each variant in order of increasing residue number. The dotted lines correspond to the "normal" range (see Supplementary Table 1). The five poorest performances (selected for display in Fig. 4 in the main paper) are highlighted in red.

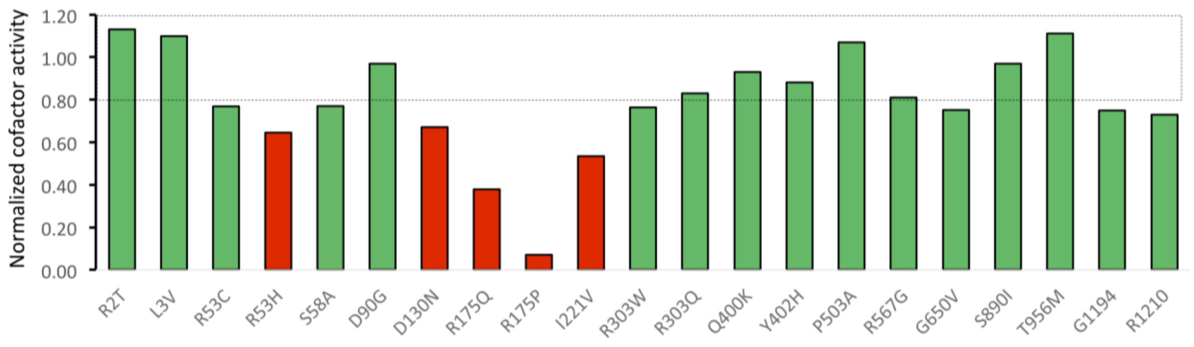

Abbreviations: ANS, 8-anilinonaphthalene-1-sulfonic acid; CA, cofactor activity; EC_{50}, half-maximal effective concentration; FH, complement factor H

Supplementary Figure 5AB. *AP immunoassay measurements for variants not severely impacted by their respective substitutions.* Representative data for (A) marginally impacted FH variants, or (B) negligibly impacted FH variants, are shown. Each panel contains overlaid plots of the quantity of sC5b-C9 detected on an AP-activating surface after that surface was exposed to FH-depleted normal human serum, as a function of the concentrations of added control FH or variant FH (error bars correspond to standard deviations for triplicate technical replicates). Plots were fitted to generate EC_{50} values. In A, the fold difference in EC_{50} value, of variant with respect to control FH, is indicated in each chart.

A

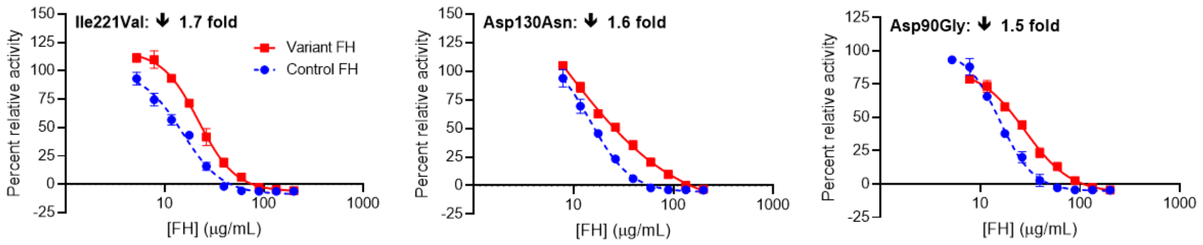

B

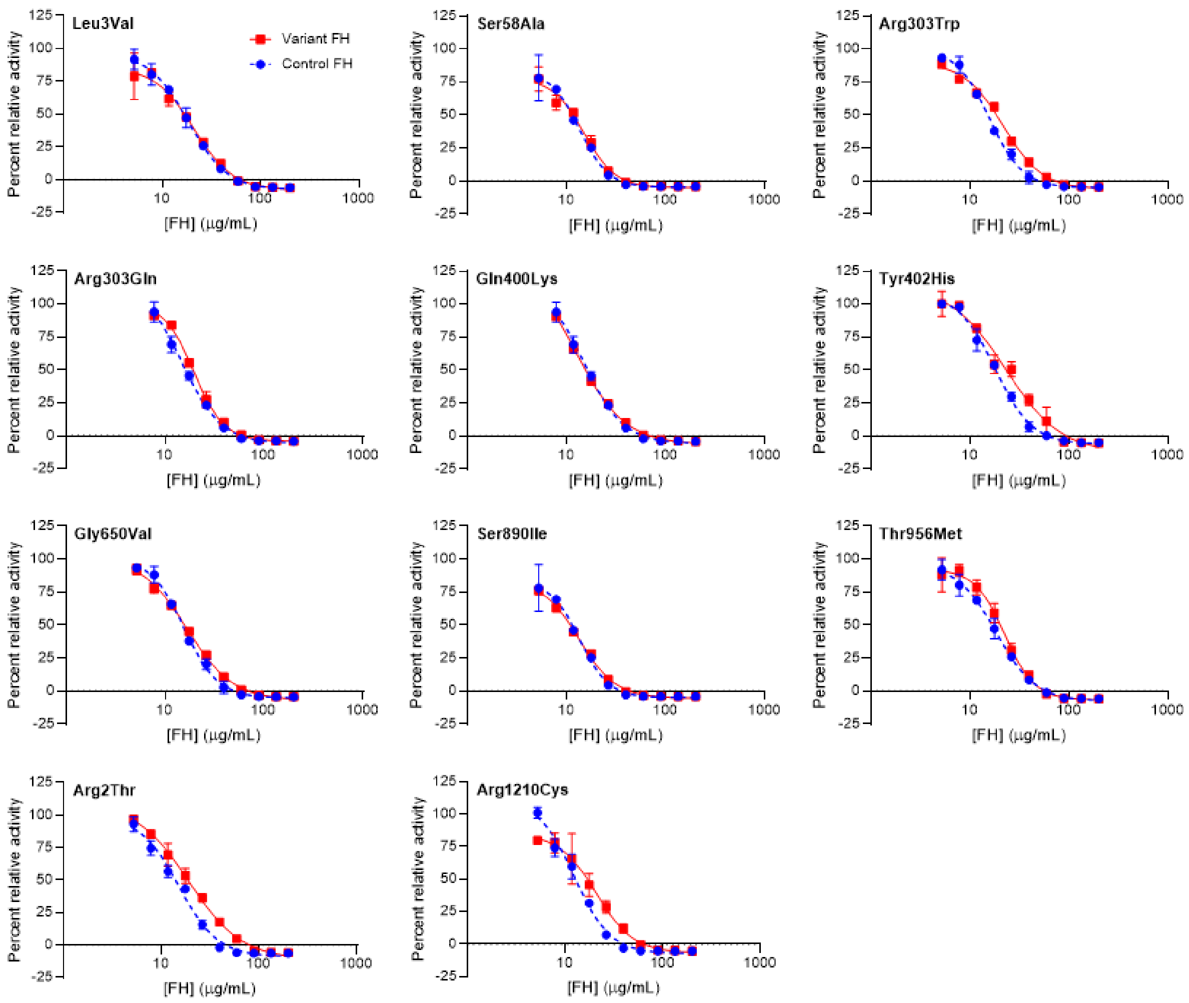

Abbreviations: AP, alternative pathway; EC_{50}, half-maximal effective concentration; FH, complement factor H

Supplementary Figure 5C. *Summary of AP-inhibition measurements for FH variants.* A column plot showing the ratios of $EC_{50}^{\text{control FH}}$/ $EC_{50}^{\text{variant FH}}$ (*i.e.*, values < 1.0 imply reduced AP inhibition) for each variant in order of increasing residue number. The dotted lines correspond to range of values for the set of three proteins (control FH, Arg2Thr and Leu3Val) produced independently but expected to have identical sequences and hence regarded as biological triplicates. The seven variants that performed least well in this assay (selected for display in Fig. 5 in the main paper) are highlighted in red.

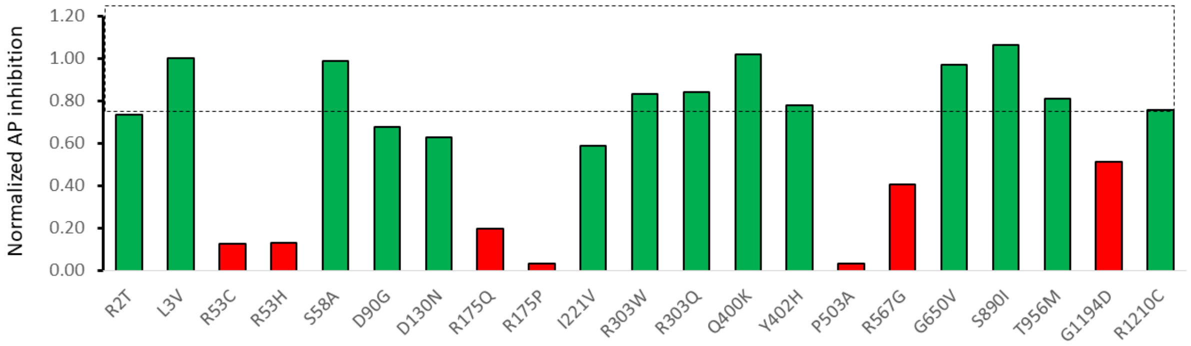

Abbreviations: AP, alternative pathway; EC_{50}, half-maximal effective concentration; FH, complement factor H

Supplementary Figure 6AB. *Hemolysis inhibition measurements for variants not severely impacted by their respective substitutions.* Representative data for (A) marginally impacted FH variants, or (B) negligibly impacted FH variants. Each panel contains overlaid plots of the extent of sheep erythrocyte hemolysis upon exposure to FH-deficient normal human serum, as a function of the concentrations of added control FH (blue, dashed) or variant FH (red, solid) (error bars: SD). Plots were fitted to generate EC_{50} values. In A, the fold difference in EC_{50} value, of variant with respect to control FH, is indicated.

A

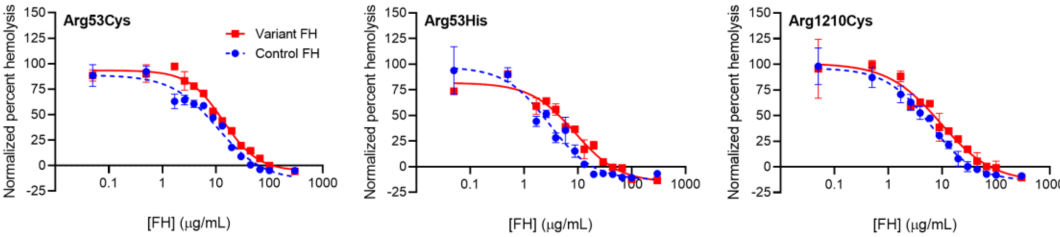

B

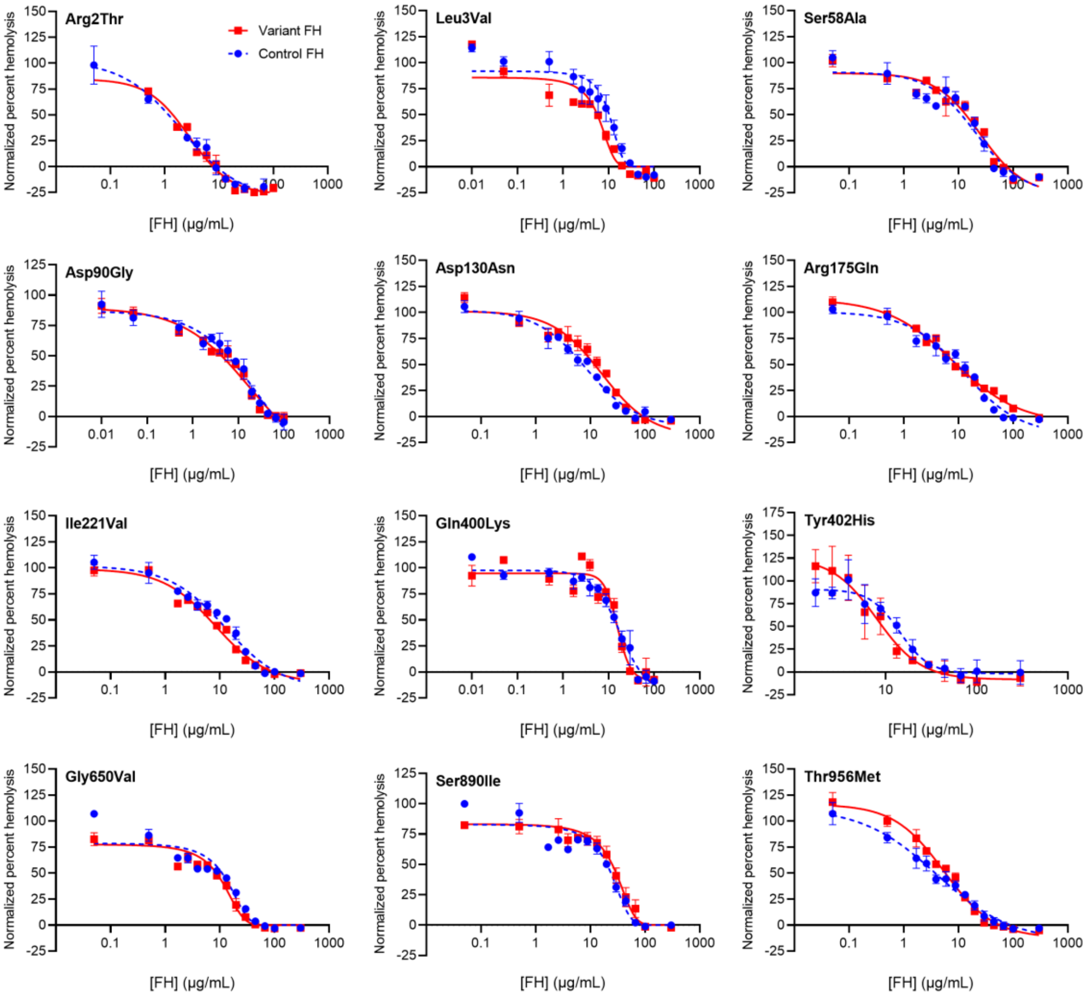

Abbreviations: EC_{50}, half-maximal effective concentration; FH, complement factor H

Supplementary Figure 7. *Correlations between assay results.* The values graphed in the column plots of Supplementary Fig. 2C, 3C, 4C and 5C are plotted against one another (red data points are for variants designated as defective) to show the extent of correlations between assay results and to identify variants that are outliers. (Top left) CA protection *versus* C3b binding; (Middle left) AP protection *versus* C3b binding; (Bottom left) DAA *versus* C3b binding; data for Arg53His, Arg53Cys, Arg175Pro are not shown as these have low activities and are off (below) the axis limits that were chosen in this case for clarity; (Top right) AP protection *versus* CA; (Middle right) DAA *versus* CA; (Bottom right) AP protection *versus* DAA

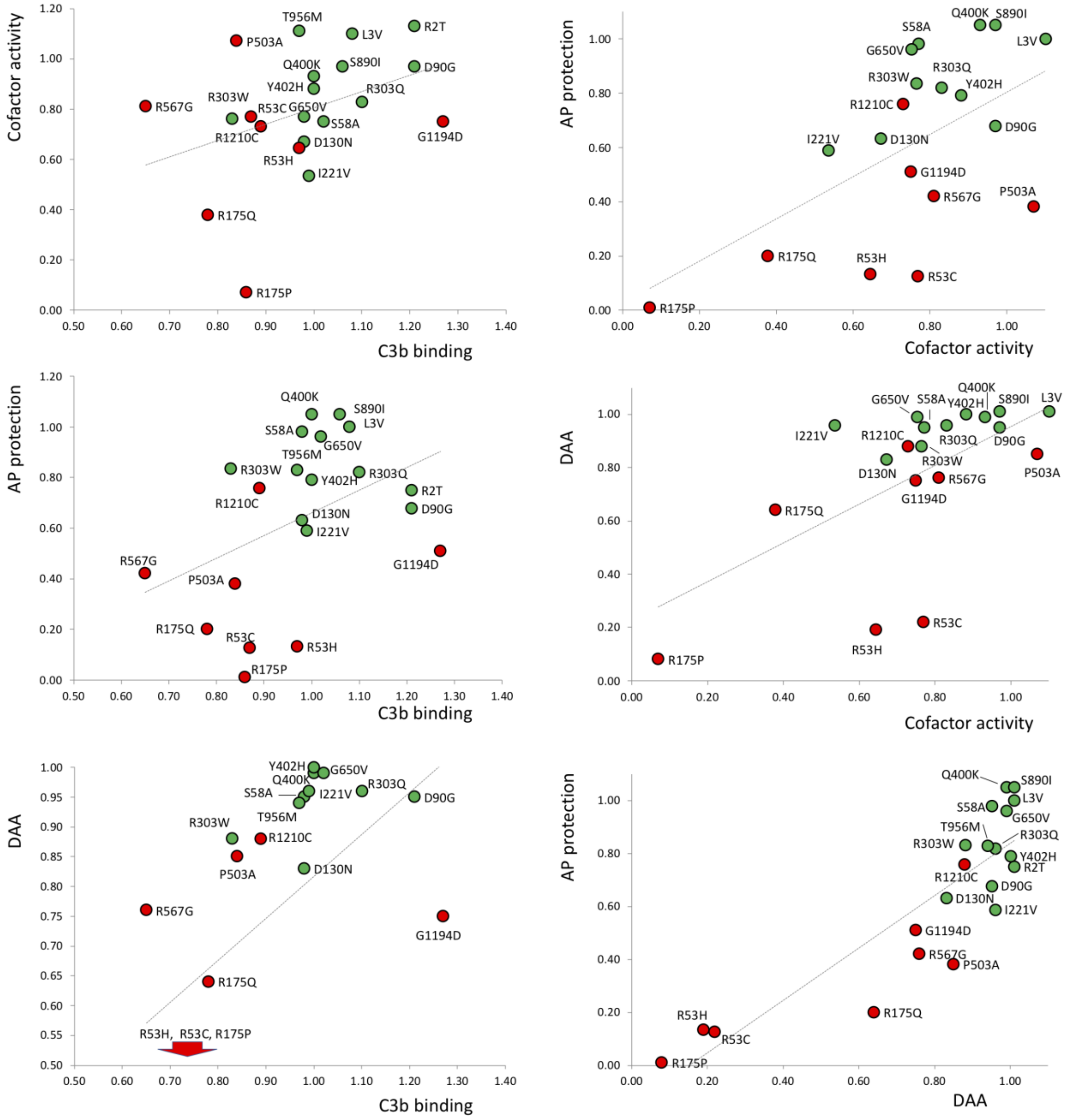

Abbreviations: AP, alternative pathway; CA, cofactor activity; C3b, complement component 3b; DAA, decay accelerating activity; FH, complement factor H

Supplementary Figure 8. Pie charts representing CESs for 21 FH variants and their locations within the CCPs of FH. The 20 CCPs of FH, in the same conformation and orientation as shown in Figure 1 (main text), are represented by ovals numbered 1–20. The modular location of the amino acid-residue substitution in each variant is indicated, along with a pie-chart style representation of its functional impact as reflected in its CES (the larger the red segment, the greater the impact).

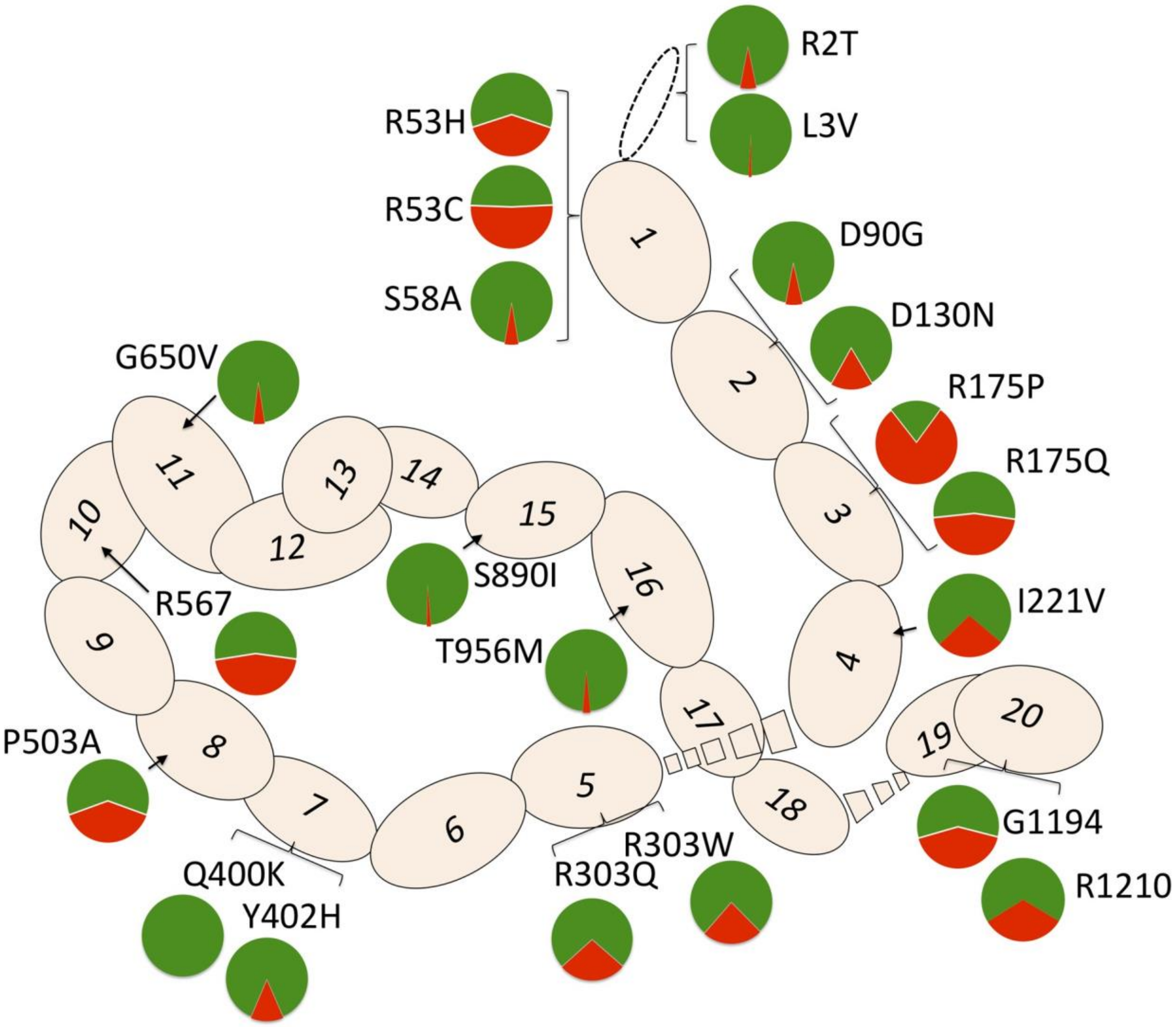

Abbreviations: CCPs, complement control protein modules; CES, combined efficacy score; FH, complement factor H

Supplementary Figure 9. Stacked plots showing the relative contributions to CES, plotted in order of ascending CES. The upper panel includes values from each of the five functional measurements and the protein yield. For the lower panel, protein yields were omitted from the CES calculations. Variants that have moved significantly within the ranking as a result are indicated by red dotted lines. Thus, the “defective" variant R53H moves still further down the ranking when considering only the functional assay data. R1210C and R303Q swap positions at the boundary between defective and marginally impacted categories. Also of some note is R2T, which

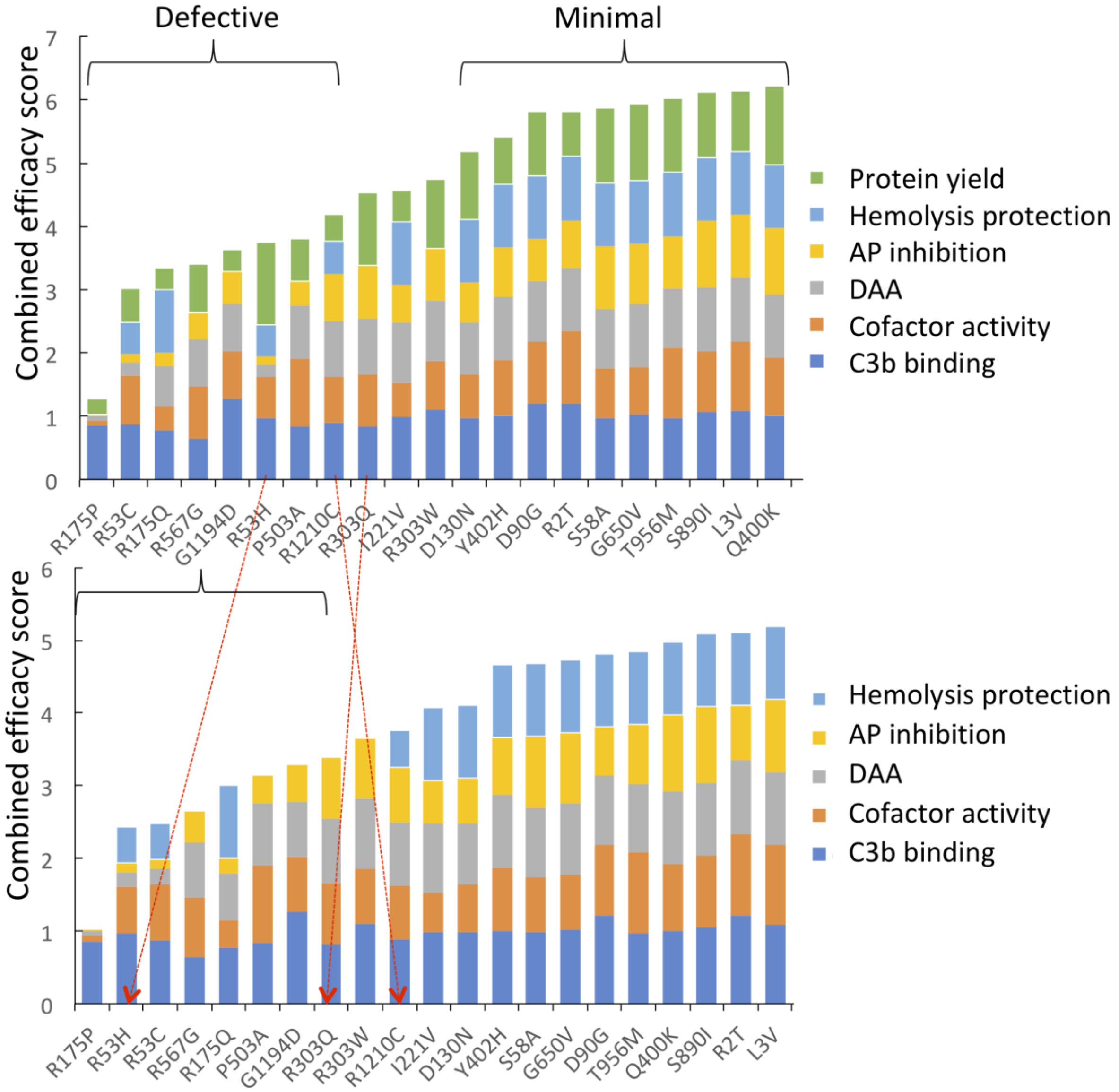

Abbreviations: AP, alternative pathway; C3b, complement component 3b; CES, combined efficacy score; DAA, decay accelerating activity

References

1. Nettleship JE, Watson PJ, Rahman-Huq N, et al. Transient expression in HEK 293 cells: an alternative to E. coli for the production of secreted and intracellular mammalian proteins. *Methods Mol Biol.* 2015;1258:209-222.
